# MicroRNAs expression dynamics reveal post-transcriptional mechanisms regulating seed development in *Phaseolus vulgaris* L.

**DOI:** 10.1038/s41438-020-00448-0

**Published:** 2021-01-10

**Authors:** José Ricardo Parreira, Michela Cappuccio, Alma Balestrazzi, Pedro Fevereiro, Susana de Sousa Araújo

**Affiliations:** 1 Instituto de Tecnologia Química e Biológica António Xavier, Universidade Nova de Lisboa, Avenida da República, 2780-157 Oeiras, Portugal; 2 Department of Biology and Biotechnology “L. Spallanzani”, University of Pavia, via Ferrata 9, 27100 Pavia, Italy; 3 InnovPlantProtect Collaborative Laboratory, Estrada de Gil Vaz, 7351-901 Elvas, Portugal; 4 Present Address: Association BLC3—Technology and Innovation Campus, Centre Bio R&D Unit, Rua Nossa Senhora da Conceição 2, Lagares da Beira, 3405‐155 Oliveira do Hospital, Portugal

**Keywords:** Seed development, Plant development

## Abstract

The knowledge on post-transcriptional regulation mechanisms implicated in seed development (SD) is still limited, particularly in one of the most consumed grain legumes, *Phaseolus vulgaris* L. We explore for the first time the miRNA expression dynamics in *P. vulgaris* developing seeds. Seventy-two known and 39 new miRNAs were found expressed in *P. vulgaris* developing seeds. Most of the miRNAs identified were more abundant at 10 and 40 days after anthesis, suggesting that late embryogenesis/early filling and desiccation were SD stages in which miRNA action is more pronounced. Degradome analysis and target prediction identified targets for 77 expressed miRNAs. While several known miRNAs were predicted to target *HD-ZIP*, *ARF*, *SPL*, and *NF-Y* transcription factors families, most of the predicted targets for new miRNAs encode for functional proteins. MiRNAs-targets expression profiles evidenced that these miRNAs could tune distinct seed developmental stages. MiRNAs more accumulated at early SD stages were implicated in regulating the end of embryogenesis, postponing the seed maturation program, storage compound synthesis and allocation. MiRNAs more accumulated at late SD stages could be implicated in seed quiescence, desiccation tolerance, and longevity with still uncovered roles in germination. The miRNAs herein described represent novel *P. vulgaris* resources with potential application in future biotechnological approaches to modulate the expression of genes implicated in legume seed traits with impact in horticultural production systems.

## Introduction

The common bean, *Phaseolus vulgaris* L., is one of the most important grain legume worldwide due to its high protein content, dietary fiber, and essential vitamins and minerals^1^. Besides, common bean immature pods are also consumed as vegetables, constituting a relevant fresh commodity with increasing world production^2^.

Seed development (SD) is a complex process that starts with a double fertilization and usually comprises three stages in orthodox seeds: embryogenesis, filling, and desiccation^3^. We described the main molecular and metabolic mechanisms underlying SD in *P. vulgaris* by analyzing proteomic and transcriptomic changes in seeds harvested at 10, 20, 30, and 40 days after anthesis (DAA)^4,5^. The 10 DAA matched the late embryogenesis, supported by evidence of high metabolic activity, including cell division and DNA synthesis. The 20 DAA matched the mid filling/maturation stage, in which the synthesis and accumulation of storage compounds was evidenced. The 30 DAA reflected the onset of seed desiccation and activation of protection mechanisms to ensure embryo quiescence and seed viability, connected with genome integrity maintenance^5^. At 40 DAA, the seed was found desiccated. Both studies highlighted a timeframe of occurring molecular and metabolic events, but the molecular mechanisms controlling the observed temporal changes in gene expression remain to be understood.

Post-transcriptional regulation mediated by microRNAs (miRNAs) controls numerous developmental processes in plants, including SD in legumes^6,7^. MiRNAs are small noncoding RNAs (sRNAs, ~21–22 nt) that act as negative post-transcriptional regulators of gene expression, either by transcript cleavage or translation repression^8^. In *P. vulgaris*, numerous studies have characterized the changes in sRNAs and miRNAs populations in different organs and growth conditions^9–15^. Evidences on the role played by some of these sRNAs in stress adaptation or symbioses were provided. As examples, pvu-miR399a was found implicated in phosphorus (P)‐deficiency signaling in common bean roots^16^, while miR319d has been implicated in nodule development^11^. In this important pulse, the few studies that have addressed seeds were focused in one or few members of miRNAs families and on specific timepoints^9,13^. Importantly, none of these studies provided comprehensive overview of miRNA abundances and repressed targets during SD. Upon availability, this knowledge can be used to support the development of approaches to improve *P. vulgaris* seed traits, including the accumulation of storage compounds or enhanced germination.

To address this gap of knowledge, a small RNA sequencing (sRNA-Seq) approach was used to identify miRNAs expressed during the main SD stages, spanning from late embryogenesis to seed desiccation. Degradome analysis and a target prediction algorithm were used to identify or predict, respectively, miRNA targets. Reverse- transcription quantitative PCR (RT-qPCR) was used to validate the sequencing results of a selected group of miRNAs and expression profiles of their targets. Using molecular network analyses, integrated overviews of the main miRNAs acting and target functional categories under miRNA regulation during SD were also provided.

## Results

### Small RNA profiles and miRNA identification in *Phaseolus vulgaris* developing seeds

**Fig. 1:**
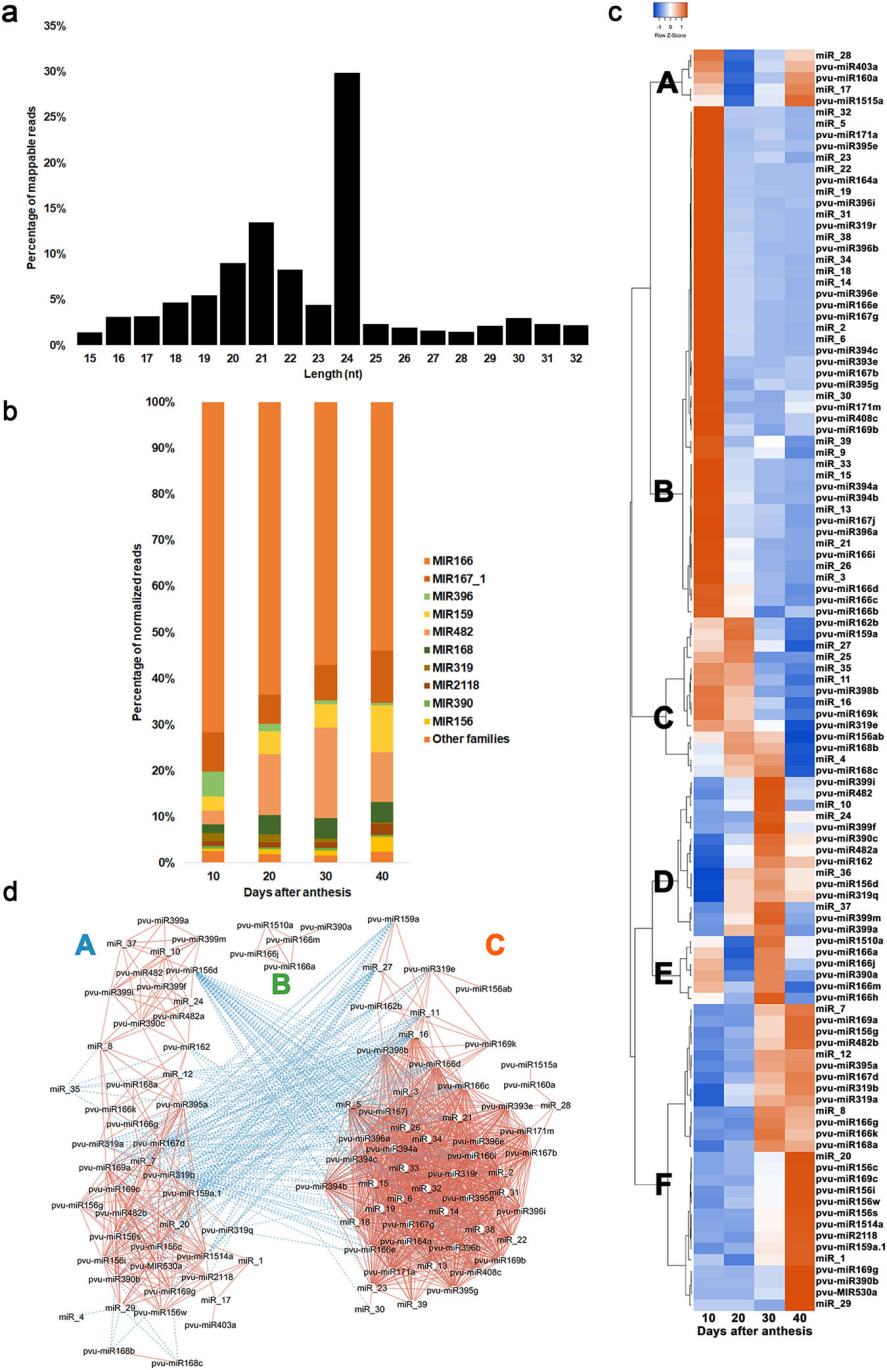
Characterization of sRNA-Seq data obtained from developing seeds of *Phaseolus vulgaris* at 10, 20, 30, and 40 days after anthesis (DAA). **a** Length distribution of mappable sRNA reads found expressed. **b** Percentage of the total amount of normalized reads for miRNA families expressed. The percentage was calculated as the sum of the average normalized reads of the miRNAs in each family, at each timepoint, divided by the total average normalized reads for all known miRNAs at that timepoint. **c** Heatmap depicting known and new miRNAs expression profiles. The heatmap was made using the average normalized read number for each miRNA at each timepoint. Pearson as distance measurement method and Complete Linkage as clustering method were applied. Letters A to F refers to the six major miRNA abundance clusters with different abundance profiles along SD. **d** Molecular interaction network of expressed miRNAs. The normalized read values from sRNA-Seq data were used on the correlation analysis. Only significant (*P*-value ≤ 0.05) correlations higher than 0.75 or lower than −0.75 are depicted. MiRNAs were used as source nodes (white circles) and the color of the lines (edges) are red for a positive correlation or blue for negative correlation

To identify miRNAs expressed during *P. vulgaris* SD, 12 independent sRNAs libraries were generated using total RNA extracts from 3 biological replicates per timepoint (10, 20, 30, and 40 DAA). A total of 230,953,396 raw reads were obtained, representing in average 19,246,116 raw reads per library (Supplementary Table 1). Of these, 175,032,524 clean reads were mappable and used for miRNA identification. The most abundant sRNA reads were 24 nt in length, representing 29.90% of the total mappable reads, followed by 21-nt RNAs with 13.5% (Fig. 1a and Supplementary Table 2).

The applied bioinformatic pipeline identified 8848 unique miRNAs, of these 141 were known miRNAs (total of 7,044,944 reads) and 8707 were considered predicted miRNAs (total of 7,507,000 reads) based on mapping against miRBase v.21 (Supplementary Table 3). The 111 miRNAs that showed a mean normalized read number higher than 100 at one SD timepoint were kept for further analysis and considered expressed in *P. vulgaris* seeds, being 51 miRNAs described as known and 60 as predicted.

With the release miRBase 22.1, these miRNAs were re-checked and a final list of 72 known and 39 new miRNAs were obtained (Tables 1 and 2 and Supplementary Tables 4 and 5). Among predicted miRNAs, only miRNAs that presented the following secondary structure prediction criteria as: (1) number of basepair (bp) in stem region (≥22), (2) free energy (dG in kcal/mol ≤ −17), (3) length of hairpin (up and down stem + terminal loop ≥56), (4) length of terminal loop (≤82), (5) number of allowed biased bulges in mature region (≤2), (6) number of basepair (bp) in mature or mature* region (≥14), (7) percentage of small RNA in stem region (pm) (≥80), and (8) minimal folding energy index (MFEI ≥ 0.80) were considered as new miRNAs (Supplementary Table 3). Additionally, MFE and secondary structure of the extended sequences (Supplementary Fig. 1) were predicted using RNAFold. MFE for the hairpin structures of the new miRNA precursors was lower than −17 kcal/mol (Supplementary Table 5). The calculated MFEI ranged from 0.8 to 2.2, with an average of 0.91 (Table 2), matching values described for miRNAs^17^. Such combined analysis showed sequences are distinct from other coding or noncoding RNAs and their secondary structures miRNAs were considered stable.

**Table 1:** Known miRNAs found expressed during seed development in *Phaseolus vulgaris*

miRNA (miRBase v21)	miRNA (miRBase v22.1)	miRNA family	miRNA ID	Sequence (5´-3´)	Length (nt)	Stem-loop strand
mtr-miR1510a-3p_1ss1CT	mtr-miR1510a-3p_1ss1CT	MIR1510	pvu-miR1510a	TGGAGGATTAGGTAAAACAAC	21	3p
gma-miR1515a	gma-miR1515a	MIR1515	pvu-miR1515a	TCATTTTGCGTGCAATGATCTG	22	5p
gma-MIR156k-p3	zma-miR156d-3p_1SS21GA_R+1	MIR156	pvu-miR156d	GCTCACTTCTCTTTCTGTCAACT	23	3p
mtr-miR156b-3p_L-1_1ss5CT	mtr-miR156c-3p_L-1		pvu-miR156c	GCTTACTCTCTATCTGTCACC	21	3p
gma-miR156a	mdm-miR156i		pvu-miR156i	TGACAGAAGAGAGTGAGCAC	20	5p
gma-miR156a_R+1	gma-miR156s		pvu-miR156s	TGACAGAAGAGAGTGAGCACT	21	5p
gma-miR156b_L+1R-1	mdm-miR156w		pvu-miR156w	TTGACAGAAGAGAGAGAGCAC	21	5p
gma-miR156c	mdm-miR156ab		pvu-miR156ab	TTGACAGAAGATAGAGAGCAC	21	5p
pvu-miR159a.1	pvu-miR159a.1	MIR159	pvu-miR159a.1	TTTGGATTGAAGGGAGCTCTA	21	3p
vun-MIR319a-p5	mdm-miR319b-5p_L+1		pvu-miR319b	TGAGCTTTCTTCAGTCCACTC	21	5p
vun-miR319b_L-2R+2	gma-miR319q		pvu-miR319q	TGGACTGAAGGGAGCTCCTTC	21	3p
gma-miR319a	gma-miR319a		pvu-miR319a	TTGGACTGAAGGGAGCTCCC	20	3p
gma-miR159a-5p_R-1	gma-miR159a-5p_R-1		pvu-miR159a	GAGCTCCTTGAAGTCCAATT	20	5p
gma-miR160a-5p	ata-miR160a-5p	MIR160	pvu-miR160a	TGCCTGGCTCCCTGTATGCCA	21	5p
gma-MIR162c-p5	stu-miR162b-5p	MIR162_1	pvu-miR162b	GGAGGCAGCGGTTCATCGATC	21	5p
gma-miR162a_R+1	vvi-miR162		pvu-miR162	TCGATAAACCTCTGCATCCAG	21	3p
mes-miR164a	mes-miR164a	MIR164	pvu-miR164a	TGGAGAAGCAGGGCACGTGCA	21	5p
pvu-miR166a_L+1R-2	gma-miR166k_L-1	MIR166	pvu-miR166g	CTCGGACCAGGCTTCATTCC	20	3p
gma-miR166a-5p_L-1R+1	gma-miR166a-5p_L-1R+1		pvu-miR166d	GAATGTTGTCTGGCTCGAGGA	21	5p
gma-miR166i-5p_R+1	gma-miR166i-5p_R+1		pvu-miR166i	GGAATGTCGTCTGGTTCGAGA	21	5p
gma-MIR166e-p5	mtr-miR166e-5p		pvu-miR166e	GGAATGTTGGCTGGCTCGAGG	21	5p
gma-miR166a-5p	aly-miR166c-5p		pvu-miR166c	GGAATGTTGTCTGGCTCGAGG	21	5p
pvu-miR166a_L+1R-1	bdi-miR166e-3p_1SS1CG		pvu-miR166m	GTCGGACCAGGCTTCATTCCC	21	5p
gma-miR166a-3p_1ss21CA	aly-miR166b-3p_1SS21CA		pvu-miR166b	TCGGACCAGGCTTCATTCCCA	21	3p
gma-miR166a-3p	pvu-miR166a		pvu-miR166a	TCGGACCAGGCTTCATTCCCC	21	3p
pvu-miR166a_1ss21CG	gma-miR166j-3p		pvu-miR166j	TCGGACCAGGCTTCATTCCCG	21	3p
pvu-MIR166a-p3	gma-miR166k_R-1		pvu-miR166k	TCTCGGACCAGGCTTCATTC	20	3p
pvu-miR166a_L+2R-2	gma-miR166k		pvu-miR166h	TCTCGGACCAGGCTTCATTCC	21	3p
ahy-miR167-3p	ptc-miR167g-3p	MIR167_1	pvu-miR167g	AGATCATGTGGCAGTTTCACC	21	3p
gma-miR167a_R-1	mdm-miR167b_R-1		pvu-miR167b	TGAAGCTGCCAGCATGATCT	20	5p
gma-miR167c_R+1	ath-miR167d		pvu-miR167d	TGAAGCTGCCAGCATGATCTGG	22	5p
gma-miR167e_R+1	mdm-miR167j		pvu-miR167j	TGAAGCTGCCAGCATGATCTTA	22	5p
gma-MIR168a-p3	ptc-miR168b-3p	MIR168	pvu-miR168b	CCCGCCTTGCATCAACTGAAT	21	3p
ath-miR168b-3p_R+1_2ss5TC10TC	aly-miR168a-3p_R+1		pvu-miR168c	CCCGCCTTGCATCAACTGAATT	22	3p
gma-miR168a	bna-miR168a		pvu-miR168a	TCGCTTGGTGCAGGTCGGGAA	21	5p
gma-miR169k	gma-miR169k	MIR169_2	pvu-miR169k	CAGCCAAGAATGACTTGCCGG	21	5p
vun-miR169	zma-miR169c-5p		pvu-miR169c	CAGCCAAGGATGACTTGCCGG	21	5p
gma-miR169a_R+1	gma-miR169a_R+1		pvu-miR169g	CAGCCAAGGATGACTTGCCGGA	22	5p
vun-MIR169-p3	zma-miR169a-3p_L+1R-1_1SS8TG		pvu-miR169b	CGGCAAGTGGTTCTTGGCTAC	21	3p
gma-miR169l-3p_L-2_1ss14TC	zma-miR169a-3p_R-1		pvu-miR169a	GGCAAGTTGTTCTTGGCTAC	20	3p
gma-miR171j-5p	ath-miR171a-5p	MIR171_1	pvu-miR171a	TATTGGCCTGGTTCACTCAGA	21	5p
gma-miR171m	gma-miR171m		pvu-miR171m	TTGAGCCGCGTCAATATCTCA	21	3p
pvu-miR2118	pvu-miR2118	MIR2118	pvu-miR2118	TTGCCGATTCCACCCATTCCTA	22	3p
mtr-miR319c-5p_L-1R+1_1ss8CT	mtr-miR319c-5p_L-1R+1_1SS8CT	MIR319	pvu-miR319r	GAGTTCTTTGCAGCCCAAAGC	21	5p
gma-miR319g_L+1R-2	vvi-miR319e		pvu-miR319e	TTTGGACTGAAGGGAGCTCCT	21	3p
gma-miR390b-5p	gma-miR390b-5p	MIR390	pvu-miR390b	AAGCTCAGGAGGGATAGCACC	21	5p
ath-miR390a-5p	ghr-miR390a		pvu-miR390a	AAGCTCAGGAGGGATAGCGCC	21	5p
ath-miR390a-3p_R-1	aly-miR390a-3p_R-1		pvu-miR390c	CGCTATCCATCCTGAGTTTC	20	3p
gma-miR393a_R+1	gma-miR393e	MIR393	pvu-miR393e	TCCAAAGGGATCGCATTGATCC	22	5p
gma-miR394a-5p_R-2	gma-miR394c-5p_R-2	MIR394	pvu-miR394c	TTGGCATTCTGTCCACCT	18	5p
gma-miR394a-5p	cpa-miR394a		pvu-miR394b	TTGGCATTCTGTCCACCTCC	20	5p
gma-miR394a-5p_R+1	vvi-miR394a_R-1_1SS21AT		pvu-miR394a	TTGGCATTCTGTCCACCTCCT	21	5p
gma-MIR395f-p5_1ss21GA	aly-miR395e-5p_2SS19TC20TA_L+1R-1	MIR395	pvu-miR395e	AGTTCCTCTGAGCACTTCACA	21	5p
gma-miR395a_1ss18AG	gma-miR395a_1ss18AG		pvu-miR395a	CTGAAGTGTTTGGGGGAGCTC	21	3p
gma-miR395d	gma-miR395g		pvu-miR395g	TGAAGTGTTTGGGGGAACTTT	21	3p
gma-miR396b-3p	mtr-miR396a-3p	MIR396	pvu-miR396b	GCTCAAGAAAGCTGTGGGAGA	21	3p
gma-miR396a-3p_L+1	sly-miR396a-3p		pvu-miR396i	GTTCAATAAAGCTGTGGGAAG	21	3p
gma-miR396a-5p	mes-miR396a		pvu-miR396a	TTCCACAGCTTTCTTGAACTG	21	5p
gma-miR396b-5p	atr-miR396e		pvu-miR396e	TTCCACAGCTTTCTTGAACTT	21	5p
gma-miR398a_L+2R-2	stu-miR398b-3p_L+1R-1	MIR398	pvu-miR398b	TTTGTGTTCTCAGGTCACCCC	21	3p
zma-miR399f-5p_1ss22AT	zma-miR399f-5p_1ss22AT	MIR399	pvu-miR399f	GGGCAACTTCTCCTTTGGCAGT	22	5p
mtr-miR399b	osa-miR399i		pvu-miR399i	TGCCAAAGGAGAGCTGCCCTG	21	3p
pvu-miR399a	pvu-miR399a		pvu-miR399a	TGCCAAAGGAGAGTTGCCCTG	21	3p
gma-miR403a	gma-miR403a	MIR403	pvu-miR403a	TTAGATTCACGCACAAACTTG	21	3p
gma-miR408a-3p	gma-miR408c-3p	MIR408	pvu-miR408c	ATGCACTGCCTCTTCCCTGGC	21	3p
vun-MIR482-p5	pvu-miR482-5p_R-1	MIR482	pvu-miR482	GGAATGGGCTGATTGGGAAGC	21	5p
gma-miR482b-3p	gma-miR482b-3p		pvu-miR482b	TCTTCCCTACACCTCCCATACC	22	3p
vun-miR482_L-2	vun-miR482_L-2		pvu-miR482a	TTCCCAATTCCGCCCATTCCTA	22	3p
PC-5p-11154_803	gma-MIR530a_R+1_1SS16AG	MIR530	pvu-MIR530a	TGCATTTGCACCTGCGCTTTG	21	5p
gma-MIR399h-p5	gma-miR399m_L+1R-1_2SS5TA20TG		pvu-miR399m	AGGGCACCTCTCTCCTGGCAG	21	5p
gma-MIR156f-p3	csi-miR156g-3p_2SS8AC12TC		pvu-miR156g	GCTCTCTCTTCCTCTGTCATC	21	3p
pvu-miR1514a	pvu-miR1514a		pvu-miR1514a	TTCATTTTGAAAATAGGCATTG	22	5p

The miRNA annotation in miRBase v21, v22.1 and the miRNA name in this study is provided. MiRNA family (Gene family) and stem-loop strand was obtained from miRBase 22.1 (for more details see Supplementary Table 4)

**Table 2:** Candidate miRNAs found expressed during seed development in *Phaseolus vulgaris*

miRNA ID	Sequence (5´-3´)	Length (nt)	MFE (kcal/mol)	MFEI
miR_1	TTGTTTTTCCTATTCCACCAAT	22	−41.8	1.2
miR_2	TTTAAGAATTTCAGTTATGC	20	−41	1.2
miR_3	TAACTGAATATTCTTAAAG	19	−41	1.2
miR_4	GCTCTCTATATTTCTGTCATC	21	−48	1
miR_5	TGCTGCTAGTTCATGGATACC	21	−83.7	1.1
miR_6	CATGTGCCCCTCTTCCCCATC	21	−55.3	0.8
miR_7	GGCAAGTTGGCCTTGGCTATA	21	−69.1	1
miR_8	AAGTAGAGTGCAGCCAAGGAT	21	−63.5	1
miR_9	TCGTCCTGAGACCACATGAGA	21	−61.4	0.9
miR_10	GGGCAATGGCTTCTTTGGCAGT	22	−59.6	0.8
miR_11	AACCTTGGTGACTAATTAGATACC	24	−83.3	2.2
miR_12	TTCTTTCAAACAGGCCCTGAG	21	−48.1	1.1
miR_13	AATAGAATTCTAGATTAGAAGATC	24	−37.9	0.8
miR_14	AGAGACCGAGACACGTCATGACGT	24	−34.1	1
miR_15	CCCGTGCGACCAAAATAAATTATT	24	−54.5	0.8
miR_16	TATGATTTCCTTTGCTTCCTC	21	−72.6	1.3
miR_17	TCTTCTCTCTATTGTCACCTT	21	−52	0.9
miR_18	CATTTGTTTCTTTCTCTCCTTATA	24	−50.7	1.2
miR_19	AATGGAGCATAGATATTGAATATA	24	−82.5	1.5
miR_20	TTAGATTTTAAAATTTGGGAC	21	−17	1.3
miR_21	TGGATGAGACGCTCATTTGAG	21	−45.5	1.1
miR_22	GACACGGACACATCATTTAAGAGA	24	−30.8	1
miR_23	AAACGAATTTTCAACGTGGACTGT	24	−35.2	0.9
miR_24	ACAAGAGGCAGAAAGTAGAGTG	22	−57.9	1
miR_25	CATCAAGATTGTATACAACTCT	22	−93.6	1.5
miR_26	CAAATGAGTATTCCATCTACA	21	−45.5	1.1
miR_27	ATGAAAATCCAGAACTCATAACTC	24	−36.4	0.9
miR_28	CTAATCAAGGAAATCACAGTAG	22	−33.5	0.8
miR_29	CGTTCCTGCATGGGGGCACCA	21	−87.2	1.1
miR_30	GCCGAGAATTGAGATCCATAACTC	24	−52.5	0.8
miR_31	AAAATAAATTATTATGGTCGTCGT	24	−54.5	0.8
miR_32	GGTTTGTGCGTGAATCTGACG	21	−44.88	1.1
miR_33	TAACTGAATATTCTTAAAGCCT	22	−41	1.2
miR_34	AAGGGTTTCTATCAGAGTTTA	21	−48.6	0.8
miR_35	TGTAAAACCCTGAATCCAAACCAT	24	−79.8	1
miR_36	CAATGAGGGCATGTTGTAGGC	21	−54.5	1
miR_37	GGGCATATCTTCTTTGGCACA	21	−56.6	0.9
miR_38	ACCAGAGTTTCCATTTTAACATAT	24	−71	1.7
miR_39	TAAGATGAAAACAAGGATACACT	23	−66.4	0.9

*MFEI* minimum folding free energy index, *MFE* minimal free energy

### Twenty-five known miRNA families were found expressed during seed development

Seventy-two known miRNAs were identified, belonging to 25 miRNA families (Table 1). MIR166 is the most represented family based on the highest percentage of reads (Fig. 1b and Supplementary Table 6), having the highest number of isoforms (11). Pvu-miR166a and pvu-miR166h are the most expressed miRNAs showing no relevant changes on their abundance (Supplementary Fig. 2). Families MIR167_1 and MIR482 also presented high read number. MIR156 and MIR169 also stood out with different abundance profiles during SD. A peak of abundance was observed at 30 DAA for MIR399 and MIR482, while on MIR396 the isoforms abundance decreases along SD (Supplementary Fig. 2 and Supplementary Table 4).

The abundance of 60 known miRNAs showed significant differences among all timepoints (analysis of variance (ANOVA) adj. *P*-value ≤ 0.05) (Supplementary Table 4). Among those, 23 were differentially expressed (DE) between 10 and 20 DAA (adj. *P*-value ≤ 0.05) and none in the other comparisons established. Between 10 and 40 DAA, 44 known miRNAs were found DE (Supplementary Fig. 3).

### Thirty-nine new miRNAs were found expressed during seed development

Thirty-nine new miRNAs were identified, 17 with length of 21 nt and the remaining with length between 19 and 24 nt (Table 2 and Supplementary Table 5). Some new miRNAs have aligned, with more mismatches than those allowed by our criteria, against known mature miRNAs/stem-loops available in miRBase v22.1 (Supplementary Table 5). These observations provide insights into the families that these miRNAs might belong to. For instance, miR_28 partially aligned to gma-miR1509a, belonging to the MIR1509 family, while miR_33 partially aligned against gma-miR1512a-5p belonging to the MIR1512 family.

The abundance of 32 out of 39 new miRNAs, showed significant differences among all timepoints (ANOVA adj. *P*-value ≤ 0.05) (Supplementary Table 5). Among those, 14 were DE between 10 and 20 DAA (adj. *P*-value ≤ 0.05) and none in the other comparisons established. Between 10 and 40 DAA, 23 new miRNAs were found DE (Supplementary Fig. 3).

### A high number of miRNAs accumulates preferentially during late embryogenesis and desiccation

Principal component analysis (PCA) conducted with all expressed miRNAs showed that the replicates of each studied stage clustered together, discriminating well the four experimental groups (Supplementary Fig. 4). Hierarchical clustering highlighted six major miRNA abundance clusters with different abundance profiles along SD (Fig. 1c). MiRNAs grouped in cluster A have high abundance at 10 and 40 DAA, while those on cluster B have the highest abundance at 10 DAA, decreasing afterwards. Cluster C grouped miRNAs with lowest abundance at 40 DAA. Cluster D groups miRNAs with highest abundance at 30 DAA, while Cluster E groups miRNAs with high abundance at 10 and 30 DAA. In cluster F, miRNAs with the highest expression at 30 and/or 40 DAA were found.

Correlation networks were made with the 106 miRNAs that presented a strong and significant correlation (*R*^2^ ≤ −0.75 and *R*^2^ ≥ +0.75, *P*-value ≤ 0.05) in their expression (Fig. 1d and Supplementary Table 7). Among others, miRNA groups A and C have inverse correlations between them. Most of group A miRNAs had the highest abundance at 40 DAA, while for group C most of the miRNA had the highest abundance at 10 DAA. Several miRNAs showed more than 50 significant correlations with other miRNAs, such as pvu-miR166d, miR_16, pvu-miR398b, pvu-miR166c and miR_26.

### Validation of sRNA sequencing data by RT-qPCR

Two known (pvu-miR399a, pvu-miR156i) and seven new DE miRNAs (miR_6, miR_11, miR_16, miR_18, miR_29, miR_33 and miR_38) were selected for RT-qPCR validation of sequencing results and to examine their expression pattern along eight SD stages studied (Supplementary Fig. 5). A strong positive correlation (0.71 ≤ *R*^2^ ≤ 0.99) between sRNA-Seq and RT-qPCR data (Supplementary Table 8) was evidenced for most miRNAs, with exception of miR_29.

### Targets for eight expressed miRNAs were validated through degradome analysis

A total of 124,993,267 raw reads were obtained from degradome sequencing, with a total of 3048 miRNA/cleaved target pairs identified after CleaveLand v4.3 analysis (Supplementary Tables 9 and 10). Of these, 133 were found in categories between 0 and 2 with a significant (*P*-value < 0.05) association between miRNA and target (Supplementary Table 10). Based on our miRNA expression threshold (100 normalized reads), only 10 miRNA:cleaved target pairs were considered in this study (Supplementary Table 11 and Supplementary Fig. 6). As examples, the *DEHYDRIN FAMILY PROTEIN (RAB18)* is a validated miR_18 target, while *DEAD BOX RNA HELICASE (PRH75)* is a validated miR_6 target.

### Targets for seventy-three miRNAs were predicted

To complement degradome information, targets for 73 miRNAs were predicted using psRNATarget (Supplementary Table 12). Several predicted targets for known miRNAs encode for transcription factors (TFs) (Supplementary Table 12). Members of the *SQUAMOSA PROMOTER BINDING PROTEIN-LIKE* (*SPL2*, *SPL4*, *SPL8*, *SPL9*, *SPL10* and *SPL12*) were predicted targets of several MIR156 members. Members of the *HOMEODOMAIN-LEUCINE ZIPPER* (*HD-ZIP*) family (*HB-8, PHB*, *REV*, and *CNA*) were predicted targets of the MIR166. *NUCLEAR FACTOR Y* family TFs (*NF-YA1, NF-YA2, NF-YA3, NF-YA8, NF-YA9* and *NF-YA10*) were described as putative targets of MIR169. *NAC* family TFs (*NAC1, NAC100, NTL9* and *CUC2*) were predicted targets of pvu-miR164a and pvu-miR1514a, while the *ABA-INDUCIBLE BASIC/HELIX-LOOP-HELIX–TYPE TRANSCRIPTION FACTOR* and *ZINC KNUCKLE* (CCHC-type) family were predicted targets of pvu-miR530a. *AUXIN RESPONSE FACTORS* (*ARF10*, *ARF16* and *ARF17*) were found targeted by pvu-miR160a, while *GRAS* TFs family were found targeted by pvu-miR171m. *SPOROCYTELESS/NOZZLE (SPL/NZZ)* was a predicted target of pvu-miR159a.1. The *HYDROXYPROLINE-RICH GLYCOPROTEIN FAMILY PROTEIN (HRGP)* and *MYB DOMAIN PROTEIN 33 (MYB33)* were predicted targets of pvu-miR319a. *HRGP* is also a predicted target of pvu-miR319q.

Transcripts implicated in phosphate metabolism, such as *PHOSPHATE 2* (*PHO2*) and *PHOSPHATE TRANSPORTER 1;1* (*PHT1;1*) were predicted targets of pvu-miR339a and pvu-miR399i. *SUCROSE-PROTON SYMPORTER 2* (*SUT1*), the *ATP SULFURYLASE 1* (*APS1*) and the *SULFATE TRANSPORTER 2;1* (*SULTR2*) were predicted targets of the MIR395 members, while the *CAROTENOID CLEAVAGE DIOXYGENASE 1* (*NCED1*) was predicted target of MIR167 members. Most of the new miRNAs identified were predicted to target transcripts encoding functional proteins (Supplementary Table 12). No miRNAs putatively targeting TFs implicated in SD such as *LEC1*, *LEC2*, *L1L*, *ABI3*, and *FUS3* were found^18^, despite the changes noticed in the abundance of these TFs along SD (Supplementary Fig. 7).

No agreement between degradome and target prediction analysis was found. Hits on the same targets identified by degradome were observed only after running psRNAtarget with less restrictive parameters (*E*-values > 5) and inputting both miRNAs and target sequences (Supplementary Table 13).

**Fig. 2:**
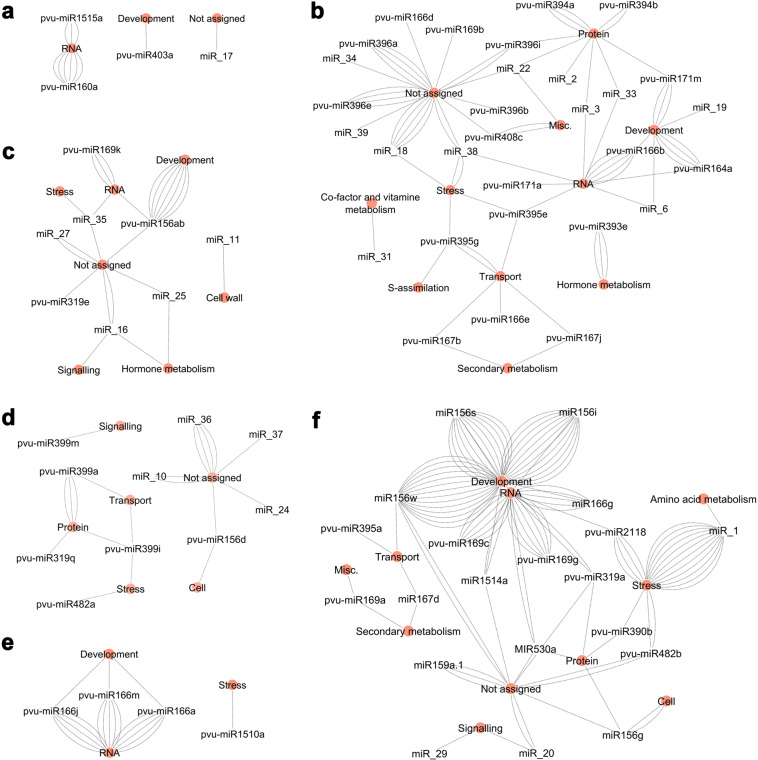
Molecular interaction network of expressed miRNAs connected to MapMan functional categories of their target genes. Each network was produced with the miRNAs from the clusters obtained using Heatmapper. **a** Cluster A. **b** Cluster B. **c** Cluster C. **d** Cluster D. **e** Cluster E. **f** Cluster F. Lines represent genes that encode the target transcripts and connect miRNAs (white circles) to MapMan functional categories of the targets (orange circles)

Molecular interaction networks were established between miRNAs grouped in the same expression cluster (Fig. 1c) and respective target MapMan functional category to investigate if any target functional category is preferentially regulated (Fig. 2 and Supplementary Tables 14 and 15). The six networks generated evidenced a relation between miRNA expression profiles and putative functions repressed by them during SD. In some cases (e.g. pvu-miR395e), the same miRNA was found to target multiple transcripts belonging to different functional categories. Focusing on the most relevant networks, the network constructed with miRNAs more accumulated at 10 DAA (Fig. 2b), shows a high number of miRNAs putatively repressing targets categorized as “Protein”, “RNA”, “Transport” and “Not assigned”. MiRNAs targeting functional categories “Hormone metabolism”, “S-assimilation” and “Secondary metabolism” were also observed, suggesting metabolic processes likely modulated on late embryogenesis/early seed filling.

The network constructed with miRNAs more accumulated at 40 DAA (Fig. 2f) showed that putatively repressed targets are mainly categorized as “Development”, “RNA” and “Stress” and to a less extent “Protein” and “Not assigned”. This network evidences that several TFs of the *SPL* family (MIR156) categorized as “Development” and *NF-YA* (MIR169) or *HD-ZIP* (MIR166) categorized both as “RNA”, as well as, several disease resistance proteins and receptors categorized as “Stress” are being potentially repressed at this stage.

### Evidences of the relevance of identified miRNAs on seed development

**Fig. 3:**
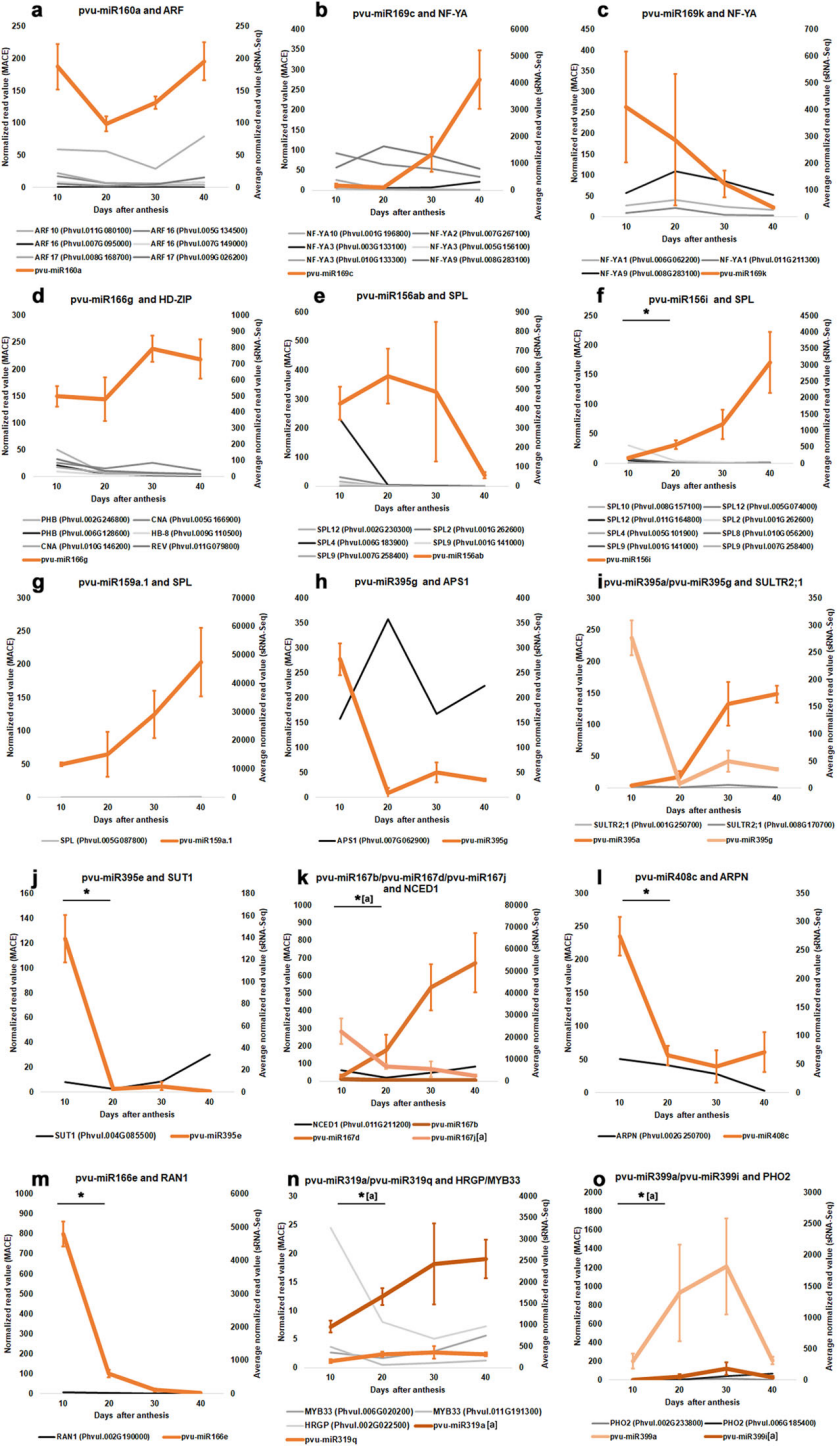
Expression profiles of selected miRNAs and targets during seed development in *Phaseolus vulgaris*. The expression profiles of the miRNAs were obtained using the average normalized read values from sRNA-Seq analysis at 10, 20, 30, and 40 days after anthesis (DAA). **a** pvu-miR160a and *ARF* members. **b** pvu-miR169c and *NF-YA* members. **c** pvu-miR169k and *NF-YA* members. **d** pvu-miR166g and *HD-ZIP* members. **e** pvu-miR156ab and *SPL* members. **f** pvu-miR156i and *SPL* members. **g** pvu-miR159a.1 and *SPL*. **h** pvu-mir385g and *APS1*. **i** pvu-miR395a/pvu-miR295g and *SULTR2;1*. **j** pvu-miR395e and *SUT1*. **k** pvu-miR167b/pvu-miR167d/pvu-miR167j and *NCED1*. **l** pvu-miR408c and *ARPN*. **m** pvu-miR166e and *RAN1*. **n** pvu-miR319a/pvu-miR319q and *HRGP/MYB33*. **o** pvu-miR399a/pvu-miR399i and *PHO2*. Error bars show the standard deviation from three sequenced biological replicates and the asterisk indicates a statistically significant difference (adj *P*-value ≤ 0.05) between consecutive timepoints. When more than one miRNA is depicted, the asterisk is followed by letters inside square brackets indicating the DE miRNA. The target expression profiles were produced using the normalized read value for target genes at the same timepoints retrieved from MACE datasets available in Parreira et al.^5^

**Fig. 4:**
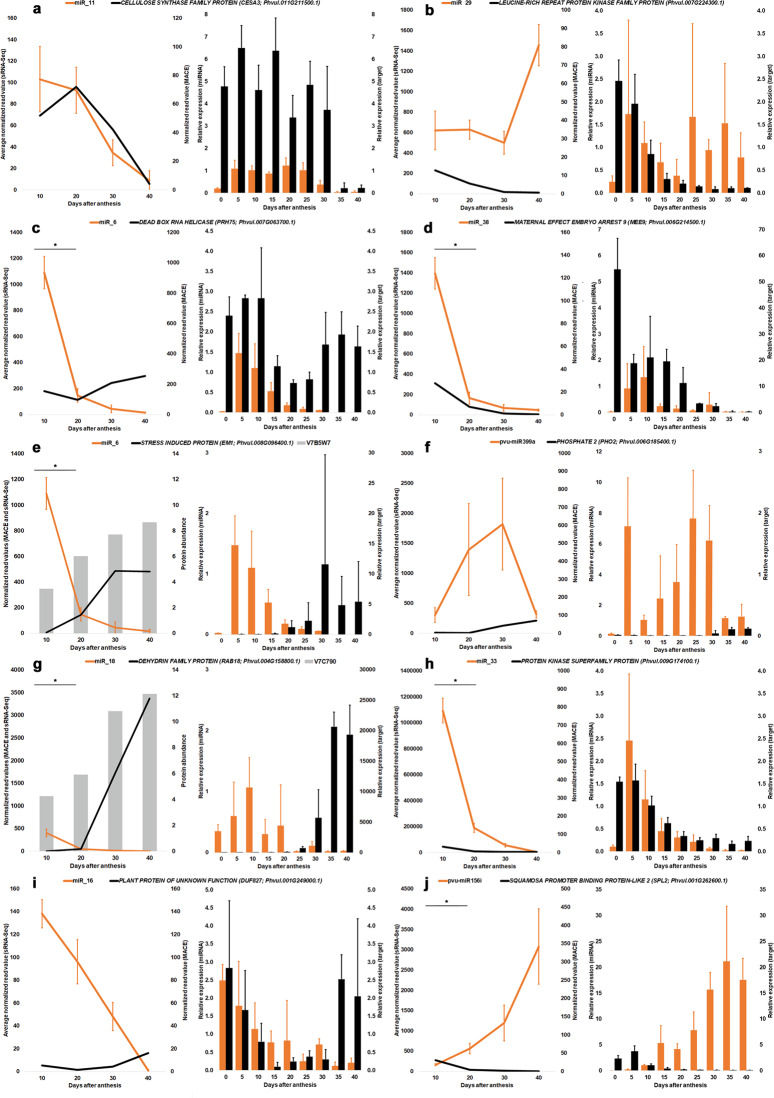
Expression profiles of selected miRNAs and targets differentially expressed during seed development in *Phaseolus vulgaris*. (Left panel) Expression profiles of the miRNAs were obtained using the average normalized read values from sRNA-Seq analysis at 10, 20, 30, and 40 days after anthesis (DAA). **a** miR_11 and *CESA3*. **b** miR_29 and *LEUCINE-RICH PROTEIN KINASE FAMILY PROTEIN*. **c** miR_6 and *PRH75*. **d** miR_38 and *MEE9*. **e** miR_6 and *EM1*. **f** pvu-miR399a and *PHO2*. **g** miR_18 and *RAB18*. **h** miR_33 and *PROTEIN KINASE SUPERFAMILY PROTEIN*. **i** miR_16 and *DUF827*. **j** pvu-miR156i and *SPL2.* Error bars show the standard deviation from three sequenced biological replicates, the asterisk indicates a statistically significant difference (adj *P*-value ≤ 0.05) between consecutive timepoints. The target expression profiles were produced using the normalized read value for target genes at the same timepoints retrieved from MACE datasets available in Parreira et al.^5^. For **e** and **g**, the protein average normalized intensity values at the same timepoints retrieved from the datasets available in Parreira et al.^4^. (Right panel) Relative expression (RT-qPCR) profiles were obtained for miRNA:target pairs in the ovary (herein defined as 0 DAA) and at subsequent eight seed development timepoints

Since miRNAs promote the cleavage/inhibition of target mRNAs, the analysis of the expression of miRNAs and their predicted/validated targets profiles can provide indirect evidence of their action. Our results evidenced timepoints, in which target downregulation mediated by miRNAs might be occurring (Figs. 3 and 4). Five MIR166 members were predicted to target *HD-ZIP* TFs such as *REV*, *PHB*, *CNA*, and *HB8* in *P. vulgaris* seeds. As one example, the high accumulation of miR166g contrasted with the relative low expression levels of its targets *CNA*, *HB-8*, *PHB*, and *REV* (Fig. 3d). Although pvu-miR166e is more accumulated at 10 DAA, its expression significantly decreases at 20 DAA (*P*-value ≤ 0.05). At the same timepoint, its degradome validated target *RAN1* shows a low abundance (Fig. 3m).

The highest abundances of miR169k and miR169c were seen at 10 DAA and 40 DAA, respectively, while abundance of their target *NF-YA9* remained low on these points (Fig. 3b, c). *NF-YA1*, another predicted miR169k target, also showed low abundances at 10 DAA.

Along SD, a progressive accumulation of pvu-miR156i was observed, while a contrasting decrease on its targets *SPL2, SPL9, SPL12* abundance was observed at the same timepoints (Figs. 3f and 4j). Similar miRNA and target expression profiles were seen for pvu-miR159.1a and pvu-miR319a during SD, while the expression of their targets was very low (Fig. 3g and Supplementary Table 4). Indeed, significant (*P*-value ≤ 0.05) increases in the pvu-miR156i (Log_2_ FC = 4.23), pvu-miR159a.1 (Log_2_ FC = 2.03), pvu-miR319a (Log_2_ FC = 1.40) and pvu-miR319q (Log_2_ FC = 1.01) abundances were observed when comparing 10 DAA values with 40 DAA ones (Supplementary Table 4).

Small RNA-Seq data showed a high accumulation of miR_6 (targeting *EM1* and *PRH75*), miR_18 (targeting *RAB18)*, miR_33 (targeting *PROTEIN KINASE SUPERFAMILY*) and miR_38 (targeting *MEE9)* at 10 DAA but decreasing significantly (*P* ≤ 0.05) at 20 DAA (Fig. 4c, d, e, g, h). The expression of their respective targets was low at 10 DAA. RT-qPCR data showed that the accumulation of miR_6, miR_33, and miR_38 occurred earlier than 10 DAA, namely at 5 DAA (Fig. 4c, e, g, h).

Numerous miRNAs targeting transcripts implicated in storage compounds and mineral allocation, signaling and homeostasis were found DE during SD. The pvu-miR395e, predicted to target *SUT1*, was strongly accumulated at 10 DAA contrasting with the low abundances seen for *SUT1* (Fig. 3j). At 20 DAA, pvu-miR395e abundances were significantly (*P*-value ≤ 0.05) lower than those observed at 10 DAA (Log_2_ FC = −5.62). Pvu-miR399a and pvu-miR399i, predicted to target *PHO2*, were found gradually accumulated until the seed start to dehydrate (30 DAA), decreasing afterwards (Fig. 3o). Indeed, for pvu-miR399i, a significant increase (*P*-value ≤ 0.05) in abundance was seen in the transition from 10 to 20 DAA (Log_2_ FC = 3.90) (Fig. 3o). Pvu-miR408c, predicted to target *ARPN*, was found strongly accumulated at 10 DAA but its abundance decreased significantly (*P* ≤ 0.05) afterwards (Fig. 3l).

Several miRNAs implicated in phytohormones metabolism have been identified. The pvu-miR160a, predicted to target *ARF10*, *ARF16*, and *ARF17* has relatively high abundance along SD, while the expression of its targets remains low (Fig. 3a). Pvu-miR167d and pvu-miR167j were found highly accumulated at 40 DAA and 10 DAA, respectively. This contrasted with the lower abundances seen for the predicted target *NCED1* (Fig. 3k). *EM1* and *RAB18* are degradome validated targets of miR_6 and miR_18, respectively. Both miRNAs are highly accumulated at 10 DAA but their abundances strongly decreased significantly (*P*-value ≤ 0.01) by comparison with 40 DAA (Log_2_ FC ≤ −6). Contrastingly, the transcript and proteomic abundances of their targets remains relatively low at 10 DAA, increasing substancially afterwards (Fig. 4e, g).

## Discussion

Herein, we provide the first comprehensive overview of miRNAs expression dynamics during *P. vulgaris* SD, spanning from late embryogenesis to seed desiccation. Seventy-two known miRNAs, belonging to 25 families, were found expressed. Thirty-nine new miRNAs were identified contributing to expand the current number of *P. vulgaris* miRNAs described. Although members of the MIR159, MIR160, MIR166, MIR399, and MIR319 have been previously described in other *P. vulgaris* tissues^10,11,13,16^, our study expands the evidences of their accumulation in developing seeds. For instance, the pvu-miR399a was found in common bean roots^16^, the pvu-miR166b in seedling leaves^14^ or the miR156 in the leaves, roots and nodules^10^. Interestingly, some of the new miRNAs identified in this study have also been reported previously in *P. vulgaris*, such as miR_32, miR_12, miR_18, and miR_19 identified as novel miRNAs by Formey et al.^11^. Notably, none of these above-mentioned studies have provided comprehensive overview of miRNA abundances and repressed targets during SD in this important pulse as the present work herein described.

**Fig. 5:**
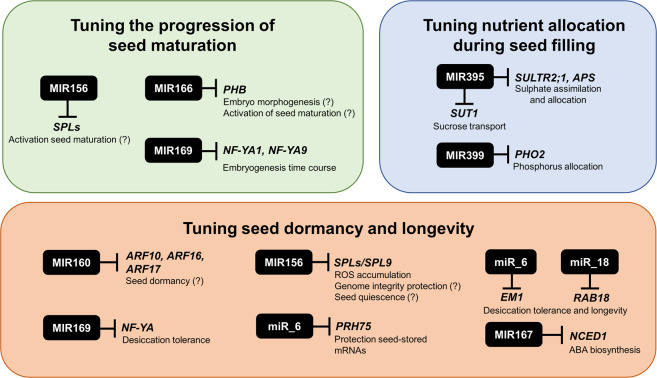
Schematic representation of the main seed developmental processes putatively regulated at post-transcriptional level by miRNAs in *Phaseolus vulgaris*. Black boxes represent the miRNAs families identified in this study, with a suggested role in tuning seed embryogenesis, maturation and dormancy. Putative miRNA targets, as well as biological functions modulated are also highlighted

Degradome analysis and target prediction identified targets for 77 expressed miRNAs. Principal component analysis (Supplementary Fig. 4), hierarchical clustering and expression correlation networks analysis (Fig. 1c, d) of miRNA abundances highlight a timeframe for miRNA accumulation, in which different miRNA groups are accumulated at these stages. Based on abundances, 10 and 40 DAA were the timepoints where the miRNAs action seems more pronounced. The miRNA correlation analysis made (Fig. 1d) evidenced two main clusters negatively correlated representing most miRNAs accumulated at early or late SD. MiRNAs/targets expression profiles provided biological evidences on relevance of these regulatory modules during SD, particularly in tuning distinct developmental stages in seeds. Nevertheless, given the complexity of regulatory networks since miRNAs can repress multiple targets, functional validation studies are needed to corroborate suggested roles in *P. vulgaris* developing seeds. This is particularly relevant to clarify if other regulatory mechanisms might be responsible for the observed downregulation of gene expression. A schematic overview of main seed developmental processes putatively regulated at post-transcriptional level by identified miRNAs and targets is provided on Fig. 5.

### Tuning the progression of seed maturation

During *P. vulgaris* SD, a global *HD-ZIP* TFs repression driven by the accumulation of MIR166 members is suggested. MIR166 was the most expressed family in this study (Fig. 1b) and is possibly regulating *REV*, *PHB*, *CNA* and *HB8* expression during all studied points. The miR166-mediated repression of *PHB*/*PHV* in early embryogenesis is involved in embryo symmetry and tissues patterning along the apical-basal axis^19^. An increased abundance of HD-ZIPs TFs at early SD was expected, since PHB is a direct activator of LEC2, which controls legume seed filling^20^. At 10 DAA, our MACE data evidenced that *PHB* abundances are already relatively low (Fig. 3d) with residual *LEC2* abundances detected (Supplementary Fig. 7). This suggests that the seed filling program activation might have occurred earlier than 10 DAA, the first sampling point for MACE and sRNA-seq. The small increase in seed size seen from 5 from 10 DAA supports this. In other species, the increase in MIR166 abundances during SD progression^21^ suggests a MIR166 role during seed maturation and quiescence. In *P. vulgaris* developing seeds, the biological relevance of the high abundance for many MIR166 members along SD is still puzzling deserving further investigation.

NF-YA, NF-YB, and NF-YC are subunits of the NF-Y heterotrimer TFs that binds to the CCAAT box in target promoter^22^. LEC1 (NF-YB9) and its closely related homolog LEC1-like (L1L, NF-YB6) are seed filing master regulators in legume and non-legume species^18,23^. A putative pvu-miR169k-mediated repression of *NF-A1* and *NF-A9* was seen at 10 DAA (Fig. 3c). NF-YA1 and NF-YA9 repression^24^ has been implicated in defining embryogenesis time course. Our data supports a similar role for this regulatory module.

MiR156-mediated repression of *SPL10* and *SPL11* during early stages of embryogenesis, prevents the precocious expression of maturation genes^25^. FUS3 triggers the expression MIR156 members implicated in the *SPL10* and *SPL11* regulation^26^. At 10 DAA, the abundances of pvu-miR156i, *SPL10* and other *SPLs*, as well as seed filling regulators *LEC2* and *FUS3* are almost residuals (Fig. 3f and Supplementary Fig. 7), suggesting that activation of the seed filling program might have occurred earlier, as described for MIR166. The majority of MIR156 members accumulates at the later SD (Supplementary Fig. 2), suggesting a global repression of *SPL* expression at 40 DAA. A MIR156 role in seed quiescence with implication on germination performance is suggested, since MIR156-*SPL* regulatory module has been implicated on the juvenile to adult transition phase of vegetative development^27^.

The miR159 mediated repression of a family of genes encoding R2R3 MYB TFs referred to as “GAMYB” or “GAMYB-like” implicated in gibberellins metabolism was found relevant to prevent abnormal vegetative development^28^. Contrary to what was described for the most of land plants, in our data *GAMYB* homologs as *MYB33* were predicted to be targets of miR139 as described for *Marchantia polymorpha*^29^. The miRNA accumulation profiles miR319/miR159 members along SD allow us to speculate that the repression of their targets is relevant during seed maturation and quiescence, but further studies are needed to clarify this.

### Tuning nutrient allocation during seed filling

We obtained indirect evidence that sucrose metabolism might be regulated at the post-transcriptional level in *P. vulgaris* seeds. In this species, the transporter *SUT1*, a sucrose/H + symporter, is highly expressed in seed coat cells and seems to be involved in sucrose efflux from the coat to the seed apoplasm^30^. Our results suggest that pvu-miR395e may repress *SUT1* expression at embryogenesis, being this repression released when the seed enters filling (Fig. 3j). In agreement with this, our previous proteomic results showed that the accumulation of proteins implicated in sugar metabolism, such as starch synthesis, and cell wall-related proteins occurs in the 10 to 20 DAA transition^4^.

MiRNAs implicated in phosphorus (P) or sulfur (S) signaling and homeostasis were identified in our seed samples. In *P. vulgaris* seeds, phytic acid is the main stored form of P, which accumulates concomitantly with dry matter accumulation^31^. While miR399-mediated *PHO2* downregulation has been implicated with P uptake on roots and translocation to shoots^32^, little is known about the MIR399 role in seeds. Our study showed that MIR399 abundances (Fig. 3o, Supplementary Table 4 and Supplementary Fig. 2) increase until 30 DAA, with significant changes observed for pvu-miR399i from 10 to 20 DAA. Such miRNA abundance profiles are in agreement with the seed filling observed in our seed samples (Supplementary Fig. 5). These results, together with the low *PHO2* expression levels observed, suggest a regulatory role for MIR399 to facilitate P translocation to the maturing seeds.

MiR395 is involved in S assimilation and allocation regulation by targeting *APS* genes and *SULTR2;1* respectively^33^. In Arabidopsis reproductive tissues, the low affinity sulfate transporter *SULTR2;1* is specifically expressed in siliques bases and veins or funiculus^34^. In agreement with this, we have found a relatively low expression of *SULTR2;1* in developing *P. vulgaris* seeds contrasting with strong MIR395 accumulation (Fig. 3i). In Arabidopsis, miR395 facilitates sulfate accumulation by targeting *APS* genes during sulfate starvation^33^. Our results highlight MIR395-mediated repression of *APS1* at 10 DAA (Fig. 3h), suggesting a role of this regulatory module on S accumulation between end of embryogenesis and early filling. In *P. vulgaris* seeds, the contents of sulfur amino are sub-optimal for human nutrition^35^. The modulation of *SULTR2;1* expression in seeds by pvu-miR395 accumulation could be a promissory approach to tune sulfate flux and enhance synthesis of S-containing storage proteins.

### Tuning seed dormancy and longevity

Auxins regulates Arabidopsis seed dormancy via a concerted action with ABA signaling pathway^36^. These authors suggested a feedback loop where ABI3-mediated repression of *MIR160B* gene, which targets *ARF10* and *ARF16*, allows the establishment of dormancy. In *P. vulgaris* seeds, the results obtained are puzzling since a potential MIR160-mediated repression of *ARF10*, *ARF16* and *ARF17* was seen along all SD (Fig. 3a). Also, *ABI3* expression levels where found increased at 30 DAA (Supplementary Fig. 7), when the seed starts to dehydrate and become dormant. Altogether, this supports that other regulatory mechanisms of gene expression might be implicated in seed dormancy.

*NCED1* encodes 9-cis-epoxycarotenoid dioxygenase, a relevant enzyme in ABA biosynthesis, whose expression was negatively correlated with ABA accumulation under water deprivation in rice^37^. Our results suggest that some members of MIR167 potentially repress *NCED1* expression (Fig. 3k), and in this way may contribute to tune ABA levels during *P. vulgaris* SD.

While it is generally accepted that ABA regulates the expression of many *LEA* genes, it has been recently shown that LEAs can be direct targets of ABI3^38^, as *EM1*^36^. LEA accumulation at late seed maturation and dehydration was implicated in embryo protection^39^. EM1 and RAB18 belong to LEA family and were found accumulated in *P. vulgaris* seeds^4^. *EM1* and *RAB18* were validated targets of miR_6 and miR_18, respectively (Supplementary Table 11). The high miRNA abundances seen at 10 DAA contrasts target transcript and protein accumulation profiles (Fig. 4e, g). In orthodox seeds, desiccation tolerance is linked to seed longevity and EM1 role on this has been recently highlighted^36^. Reduced *RAB18* abundance was implicated in a decrease in seed longevity^40^. Also, the existence of DRE and ABRE cis-acting elements in the upstream region some MIR169 members targeting *NF-YA* suggests a role in desiccation tolerance^41^. Indeed, an upregulation of several MIR169 members under drought stress was observed in soybean^42^. In wheat, *NF-YA1* decreases expression in drought stressed leaves^43^. Based on some MIR169 members accumulation profile (Supplementary Table 4), it would be interesting to further investigate if *NF-YA* repression at later SD stages represents an adaptative seed response to desiccation with impact on seed viability.

Other mechanisms implicated in seed viability and posterior germination traits seem to be regulated at the post-transcriptional level, which includes the mechanisms to cope with oxidative damage. As one example, the MIR156/*SPL9* regulatory module was implicated in the regulation of ROS accumulation, since miR156 overexpression mutants had ROS accumulation suppressed^44^. During seed filling and dehydration of *P. vulgaris* seeds, we evidenced the occurrence of oxidative damage^4^ and its effects on the maintenance of embryo genome integrity^5^. Members of the MIR156 increase expression throughout SD (Supplementary Fig. 2). For pvu-miR156i, this contrasts with the lower abundances of its predicted *SPL9* target (Fig. 3f). This suggests that MIR156/*SPL9* regulatory module can contribute to tune the ROS levels during SD and indirectly contribute to the maintenance of genome integrity. DEAD-box RNA helicases play various roles in RNA metabolism including among others ribosome biogenesis, mRNA splicing and translation^45^. Degradome analysis (Supplementary Table 11) validated *PRH75*, a nucleolar DEAD-box RNA helicase^46^, as miR_6 target. MiR_6 expression is triggered on the onset of SD since expression level was residual on ovary tissues (Fig. 4c). The contrasting miRNA/target accumulation profiles at 10 DAA, suggest a miR_6-mediated regulation of *PRH75* expression at late embryogenesis. This observation is intriguing since mutations in *PRH75* are lethal during embryogenesis in Arabidopsis^47^. During seed dormancy induction, seed-stored mRNAs for germination seem to be protected through their association with ribosome complexes that can include RNA binding proteins (RBP), such a DEAD-BOX RNA proteins^48^. A potential PHR75 involvement in this mechanism could be suggested, based on its accumulation at later stages of SD.

## Conclusion

We have identified 72 known and 39 new miRNAs expressed in the developing seeds of *P. vulgaris*. Degradome analysis and target prediction identified targets for 77 expressed miRNAs. Several transcription factor families, such as *HD-ZIP*, *ARF*, *SPL* and *NF-Y*, were identified as targets of known miRNAs, while most of new miRNAs targets were predicted to encode for functional proteins. Based on abundances, late embryogenesis/early filling (10 DAA) and desiccation (40 DAA) where stages in which miRNA action seems more pronounced. Altogether, the results support that these miRNAs tune distinct seed developmental stages. They were found implicated on controlling embryogenesis time course, postponement of seed filing and maturation program, seed desiccation tolerance and longevity, with still uncovered roles on germination. The miRNAs herein described represent novel resources with potential application in future biotechnological approaches to modulate the expression of genes implicated in legume seed traits, including storage compound accumulation or seed viability. They could be exploited, even in combination, to simultaneously target more than one seed trait and be eventually translated to other agronomically relevant legumes with impact in agricultural and horticultural production systems.

## Materials and methods

### Plant material

*P. vulgaris* genotype SER16 seeds were germinated as described^4^. Seedlings were transferred to watered vermiculite trays and maintained in a growth chamber under the following conditions: 50% relative humidity, photoperiod of 16/8 h, at 25/18 °C day/night temperature, with an average light intensity of 300 µmol/m/s. One week later, seedlings were transferred to 2.5 L pots with a (2:1:1) mixture standard “terra de Montemor” commercial soil (Horto do Campo Grande, Lisboa, Portugal), peat and vermiculite, kept on above environmental conditions and watered 3 times/week. Flowers were tagged, ovaries were sampled before anthesis (0 days) and seeds were sampled at 5, 10, 15, 20, 25, 30, 35, and 40 DAA. Two groups of samples were obtained, one used to measure seed length, fresh and dry weight to characterize the SD process and another immediately frozen in liquid nitrogen and stored at −80 °C.

### Total RNA isolation

Frozen ovaries and seeds were ground to a fine powder in liquid nitrogen using a mortar and pestle. RNA isolation was conducted as described^5^ with few modifications as the inclusion of 2% β-mercaptoethanol (v/v) on RNA extraction buffer and purifications were performed first with phenol: chloroform:isoamyl alcohol (25:24:1), followed by another with chloroform: isoamyl alcohol (24:1). Total RNA was overnight precipitated (−20 °C) by adding 6.7 µL of 3 M Sodium acetate (pH = 5.2) per 100 µL recovered and two volumes of −20 °C absolute ethanol. The Ambion® TURBO™ DNase (Life Technologies, Carlsbad, CA, U.S.A) was used to remove DNA contamination. RNA quality was assessed using a NanoDrop^tm^ 2000c Spectrophotometer (Thermo Fisher Scientific Inc., Waltham). RNA purity was estimated based on the A260/280 and A260/230 ratios absorbance ratios and was ~2 before DNAse treatment. Qubit® 2.0 Fluorometer (Thermo Fisher Scientific Inc.) with RNA BR Assay Kit was used to quantify the RNA. Extracted RNA integrity was assessed by electrophoresis in a 2.0% agarose gel. DNA contamination absence was verified by standard PCR and samples were stored at −80 °C.

### sRNA libraries construction and high-throughput sequencing

Twelve small RNA (sRNA) libraries were constructed from three biological replicates at 10, 20, 30, and 40 DAA. Each biological replicate represents seed samples collected from an independent potted plant. Libraries construction and sequencing was performed by LC Sciences (Houston, Texas, USA). sRNA libraries were generated using the Illumina Truseq™ Small RNA Preparation kit (Illumina, San Diego, USA) following the Illumina’s TruSeq™ Small RNA Sample Preparation Guide (15004197C, Illumina Inc., Part #1004239 Rev. A, 2008; Catalog # RS-930-1012, Part # 15004197 Rev. B, January 2011). Cluster generation was performed on Illumina’s Cluster Station using the purified cDNA libraries. Sequencing was performed using an Illumina Genome Analyzer IIx. Illumina’s Sequencing Control Studio (Version 2.8) was used to obtain the raw sequencing reads (40 nts), following real-time sequencing image analysis and base-calling by Illumina’s Real-Time Analysis (Version 1.8.70).

### sRNA data analysis and identification of known and novel miRNAs

The sequencing data was analyzed using the ACGT101-miR v4.2 (LC Sciences)^49,50^. This software sorts raw sequencing reads into unique families, removing adapter dimers, low complexity sequences, junk and repeats. Additionally, it also removes sequences mapped against reference database files for other noncoding RNAs (RFam database; http://rfam.janelia.org, repetitive sequences (Repbase; http://www.girinst.org/repbase) or mRNAs (*P. vulgaris* v2.1, Phytozome v12.0; DOE-JGI and USDA-NIFA, http://phytozome.jgi.doe.gov/). A detailed description of the pipeline and criteria applied to identify miRNAs and predict their secondary structure can be found on Supplementary Table 3.

The unique sequences between 15 and 32 bases were mapped to plant pre-miRNAs available in miRBase v21.0^51^ to identify known and novel miRNAs. The sequences that mapped to *P. vulgaris* miRNAs/pre-miRNAs, which also mapped against the *P. vulgaris* genome, were identified as known miRNAs. Additionally, sequences that mapped to other plant pre-miRNAs and these pre-miRNAs further mapped to *P. vulgaris* genome were also considered known miRNAs. The remaining sequences that aligned against *P. vulgaris* genome and present hairpin structure that contains the sequences were considered predicted miRNAs. Known and predicted miRNAs were filtered by the mean normalized read number. Those with  ≥100 in at least one timepoint were considered expressed and kept for further analysis. RNAfold soſtware (http://rna.tbi.univie.ac.at/cgi-bin/RNAfold.cgi) was additionally used on provided extended miRNA sequences to corroborate the potential of selected new miRNAs to form a stable hairpin^52^.

With the release of miRBase v22.1 (http://www.mirbase.org), the sequences of all selected miRNAs were re-checked, filtering by a maximum of two mismatches between the obtained miRNAs and mature miRNAs in miRBase v22.1. This was done with the purpose to clarify if, for example, any of the previously predicted new miRNAs (miRBase v21) has been already described in the latest miRBase release. Mismatches were restricted to a maximum of one mismatch in the 5ʹ region, or 2 in the 3ʹ region, and no mismatches in position 10 and 11 nt. Also, restriction was assumed for overhangs with >2 nucleotides. Valid miRNAs sequences mapping to a known precursor hairpin opposite to the annotated mature miRNA were considered novel 5p- or 3p-derived miRNA candidates. To ease their description, miRNAs within the known group were denominated as “pvu-” followed by the specific miRNA name described in miRBase. The remaining sequences are described as “miR” for new miRNAs.

An ANOVA followed by a pairwise mean comparisons (*t*-test) were applied on normalized read counts to assess differential miRNA expression, using a similar approach as described in other studies^53,54^. A Benjamini–Hochberg correction was applied to the analysis made. A hierarchical clustering analysis was performed in Heatmapper (http://www.heatmapper.ca). A principal component analysis (PCA) was performed using standardized (*Z*-score) miRNAs normalized read values as variables. Additionally, a regression analysis was conducted on the same variables to extract significant correlations. Only correlations with a *P*-value ≤ 0.05 and *r* = Pearson correlation above 0.75 or below −0.75 were considered. PCA and product-moment correlation analyses were conducted using Statistica, version 6 (Statsoft).

### Degradome sequencing

Four qualitative degradome libraries were constructed for 10, 20, 30, and 40 DAA samples by LC Sciences to identify miRNA target transcripts. Each library consists of an equimolar pool of total RNA from three biological replicates per timepoint. Sequencing was performed on the Illumina HiSeq 2500. The data was analyzed using the ACGT101-DGD (LCSciences- https://www.lcsciences.com/documents/sample_data/degradome_sequencing/DGD_html_report_DEMO.html) pipeline. Raw sequencing reads were obtained using Cutadapt^55^ and in-house perl scripts were used to remove adaptors and low quality reads. CleaveLand v4.3^56^ was used to identify potentially cleaved targets from degradome sequencing. The degradome reads were mapped to the *P. vulgaris* mRNA (Phytozome v.12). CleaveLand classifies the targets into five categories according to degradome sequencing and cleaved sequences abundance relative to the overall profile of degradome tags matching the target. Only targets within categories 0, 1, and 2 with T plot *P*-value < 0.05 and whose miRNAs were found expressed in our datasets were kept for discussion. The target annotation information was taken from *Phaseolus vulgaris* v2.1 reference genome.

### Target prediction and bioinformatic analysis

Mature sequences for all expressed miRNAs were aligned against the target transcript library of *P. vulgaris* 442_v2.1 using the web-based Plant small RNA target server (psRNATarget; http://plantgrn.noble.org/psRNATarget). Default parameters for Schema V2 (2017 release) were used, except the maximum expectation value (*E*) set to 2, to lower false-positive prediction. BLASTN (https://blast.ncbi.nlm.nih.gov/) was used on target coding sequences that presented no annotation in *P. vulgaris*. Megablast algorithm was used against the Nucleotide collection (nr/nt) database, in *Viridiplanta*e. Results with Query cover > 80% and *E*-value < 2e^−39^ were selected.

For functional categorization, coding sequences from degradome and predicted target transcripts were obtained using BioMart (Phytozome v.12, *P. vulgaris* v2.1). These sequences were used to create a mapping file for the Mercator pipeline (http://mapman.gabipd.org/app/mercator) to perform a MapMan functional categorization analysis^57^.

Cytoscape^58^ software (Version 3.7.1) was used to visualize molecular interaction networks made between expressed miRNAs and their target functional categories. MiRNAs were used as source nodes, MapMan functional category as target nodes and, to ease visualization, genes that encode target transcripts are edges connecting the nodes. MiRNAs with no predicted target identified were not included.

### Reverse-transcription quantitative PCR

Two known and seven new miRNAs and their respective targets were selected for qRT-PCR validation in samples at 0, 5, 10, 15, 20, 25, 30, 35, 40 DAA. Three biological replicates, from independent plants, were used. The two-tailed RT-qPCR method was used to quantify the miRNA expression^59^. Specific miRNAs forward, reverse and two-tailed RT primers were designed following published guidelines (Supplementary Table 16). The two-tailed RT reaction was performed using qScript Flex cDNA Kit (QuantaBio, Beverly, USA) in multiplex, whenever possible. Briefly, 110 ng RNA was used per reaction with 0.1 μM of each primer, in an adjusted total volume of 10 μL. The RT-reaction was run in a T100™ Thermal Cycler (BioRad, Hercules,CA, USA) following the published protocol^59^ conditions: 25 °C for 45 min, followed by 85 °C for 5 min.

The expression profile of ten targets was quantified by RT-qPCR (Supplementary Table 17). Each primer pair specificity was examined via Primer-BLAST (https://www.ncbi.nlm.nih.gov/tools/primer-blast/) and melting curves obtained during the assay. The RT reaction was performed with 400 ng RNA using ImProm-II™ Reverse Transcriptase (Promega, Madison, USA) in a T100™ Thermal Cycler, following the manufacturer’s instructions.

Both miRNA and target qPCR reactions were run on PikoReal Real-Time PCR System (Thermo Fisher Scientific Inc., Waltham, MA, U.S.A.). The reactions were performed in a total of 10 μL using PerfeCTa SYBR® Green SuperMix (QuantaBio, Beverly, USA) containing 1 μM of each primer in the miRNA assays, and 0.2 μM in the target assays. Reactions were performed onto same samples for both assays. Efficiency of the reactions was calculated using LinRegPCR^60^ and Pfaffl method was used to calculate relative expression^61^. Pvu-miR166n and pvu-miR1510b were used as normalizers for miRNAs qPCRs, while the eukaryotic Release Factor 1 (*eRF1*) family protein (*PEL1*) and RING/U-box superfamily protein (*XERICO*) for target qPCRs.

### Target expression analysis

The expression profile of selected predicted or degradome validated targets was established by mining Massive Analysis of cDNA Ends (MACE) datasets (NCBI Sequence Read Archive accessions: SRR6466368, SRR6466367, SRR6466366, and SRR6466365) previously described^5^ and proteomic datasets (PRIDE: PXD002254) previously described^4^. The transcriptomic and proteomic data was produced from seed samples collected at the same timepoints herein studied and come from independent experiments.

## Supplementary information

Supplementary Figures 1 to 7

Supplementary Tables 1 - 17

## Data Availability

The sRNA-Seq raw data and degradome raw data have been deposited in the NCBI Sequence Read Archive (SRA) under the accession PRJNA576378, submissions SUB6402031 and SUB6413084 respectively.
