# Supplementary material for: MicroRNAs expression dynamics reveal post-transcriptional mechanisms regulating seed development in Phaseolus vulgaris L
Source: Hortic Res. 2021 Jan 10;8:18. doi: 10.1038/s41438-020-00448-0 (PMC7804330; doi:10.1038/s41438-020-00448-0)
Supplement: Supplementary file 1 — Supplementary Figures 1 to 7 [file 41438_2020_448_MOESM1_ESM.pdf]

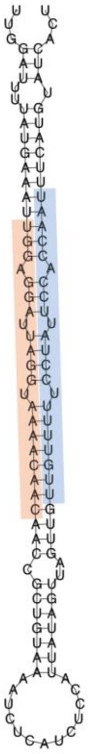

miR_1

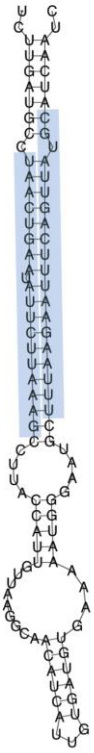

miR_2 and miR_3

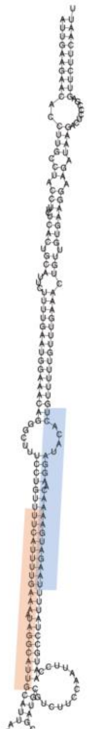

miR_39

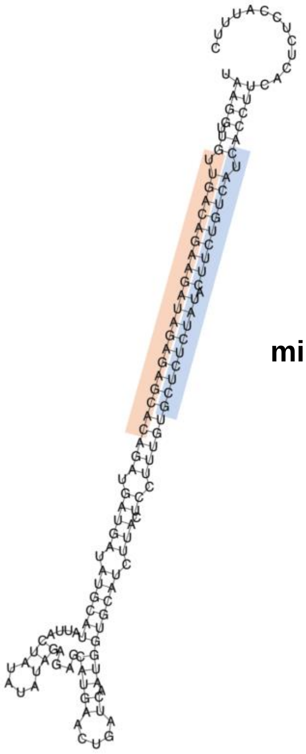

miR_4

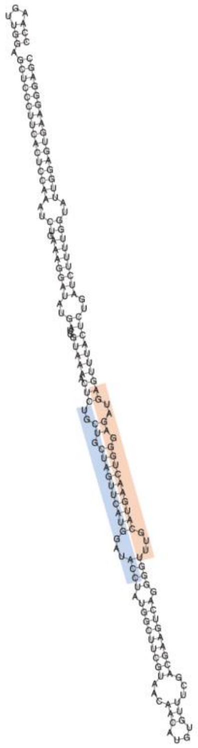

miR_5

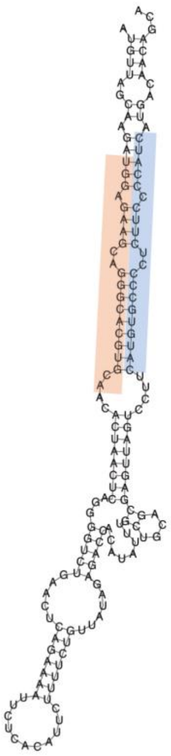

miR_6

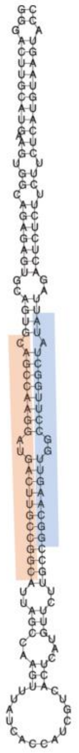

miR_7

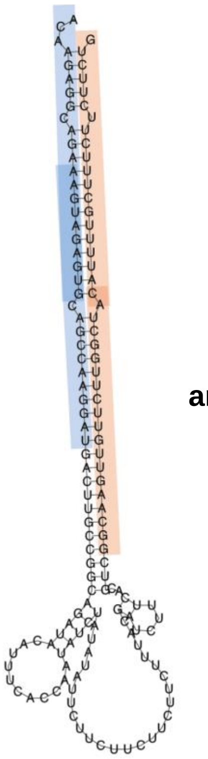

miR_24 and miR_8

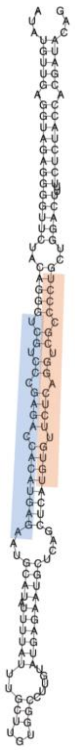

miR_9

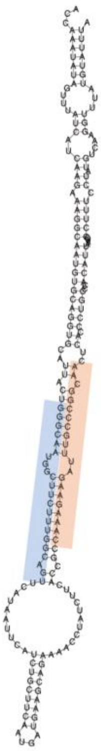

miR_10

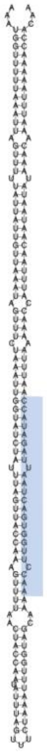

miR_11

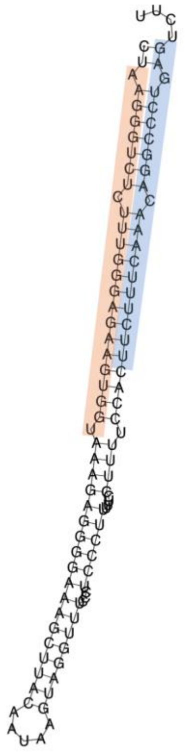

miR_12

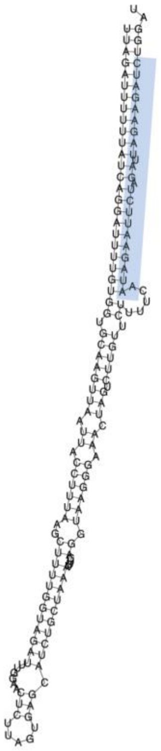

miR_13

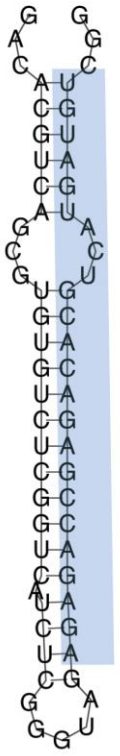

miR_14

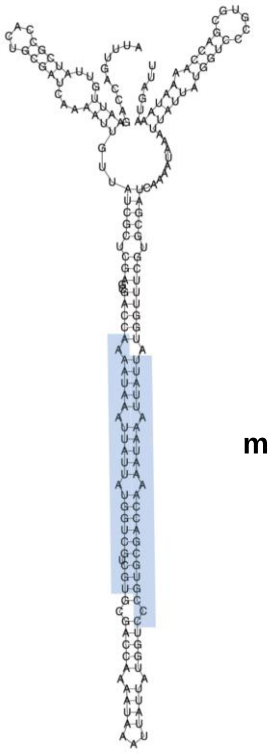

miR_15 and miR_31

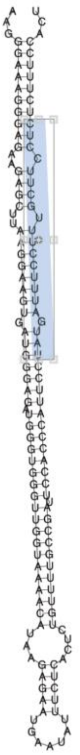

miR_16

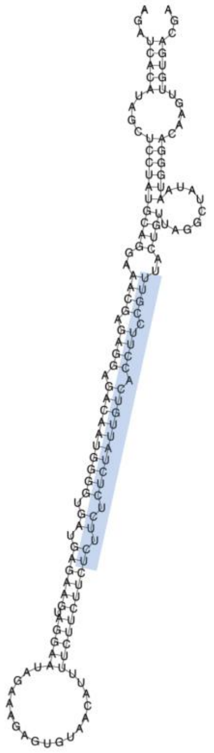

miR_17

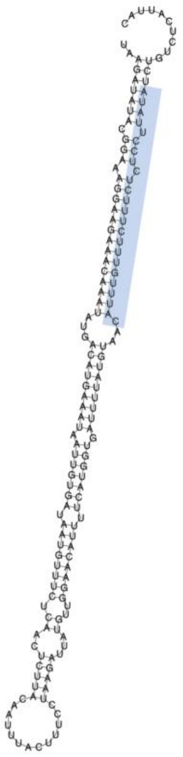

miR_18

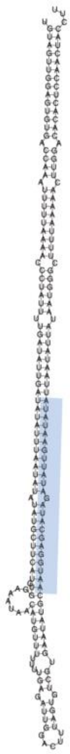

miR_19

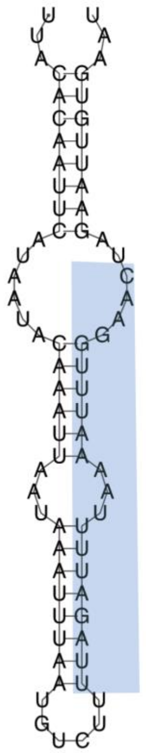

miR_20

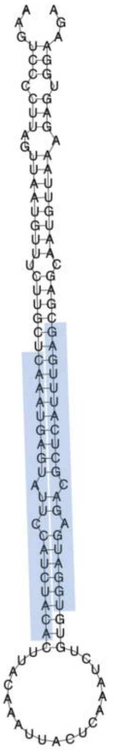

miR_21 and miR_26

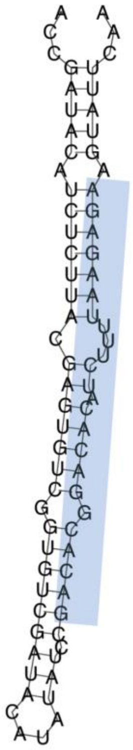

miR_22

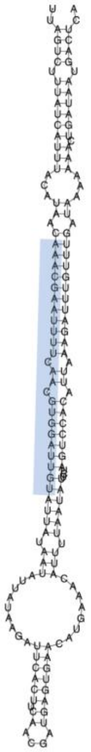

miR_23

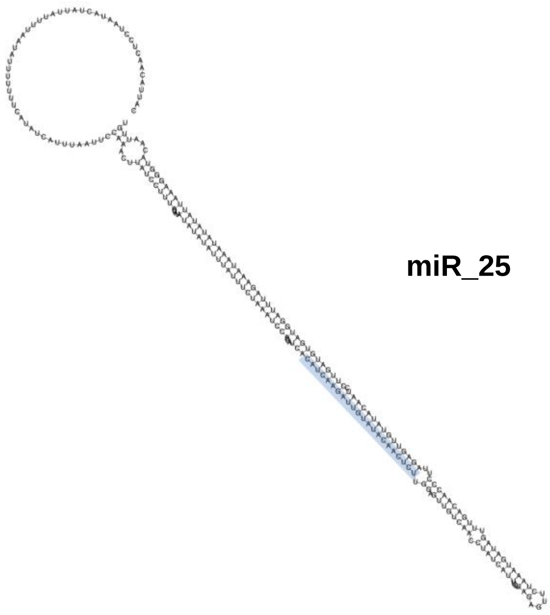

miR_25

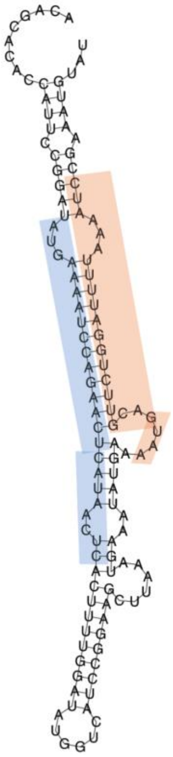

miR_27

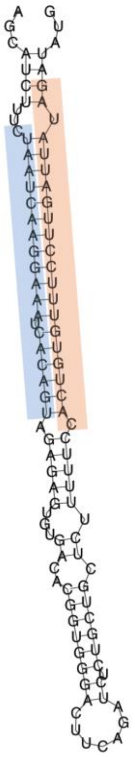

miR_28

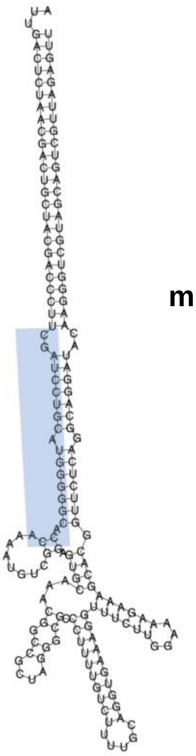

miR_29

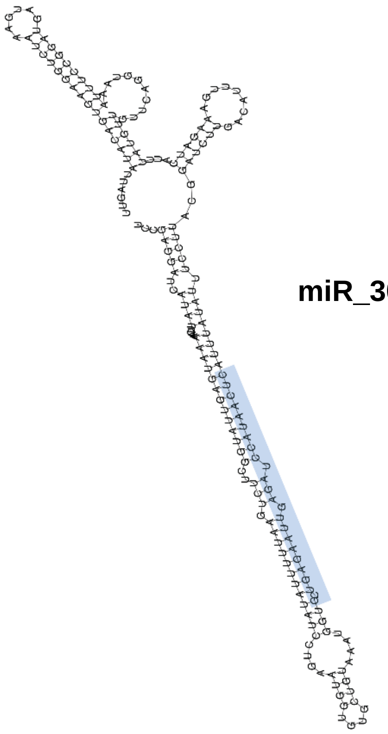

miR_30

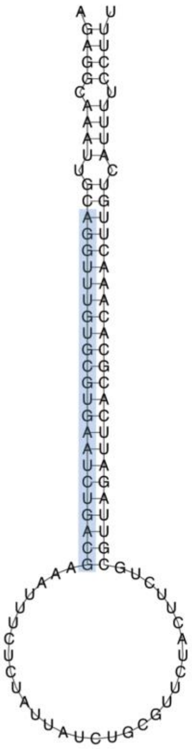

miR_32

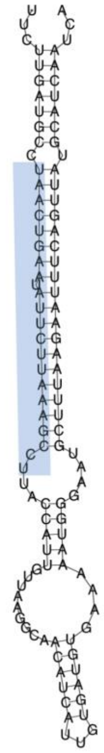

miR_33

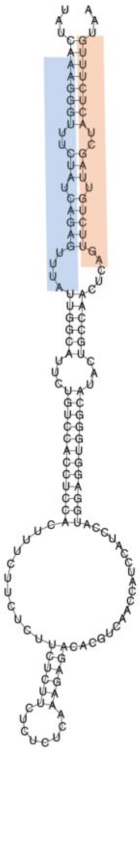

miR_34

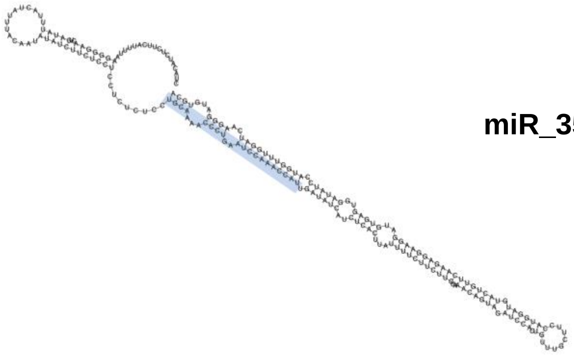

miR_35

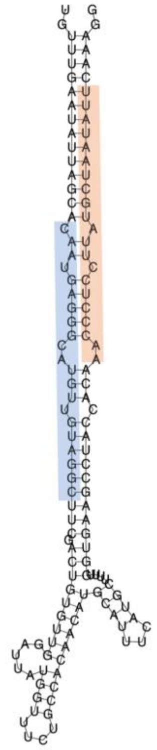

miR_36

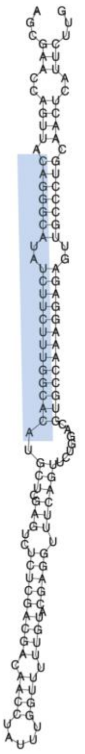

miR_37

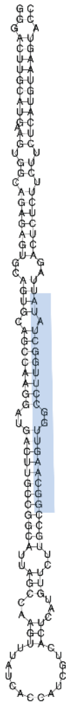

miR_38

Supplementary Fig. 1: Secondary (hairpin) structure of extended sequences for the candidate miRNAs identified in the study were obtained using RNAFold (http://rna.tbi.univie.ac.at//cgi-bin/RNAWebSuite/RNAfold.cgi). The calculated Minimal Folding Energy (MFE) of the new miRNA precursors was lower than -17/kcal/mol. The sequence of the mature miRNA is highlighted in blue, while the sequence of the mature miRNA* is highlighted in orange, if found in our sequencing data.

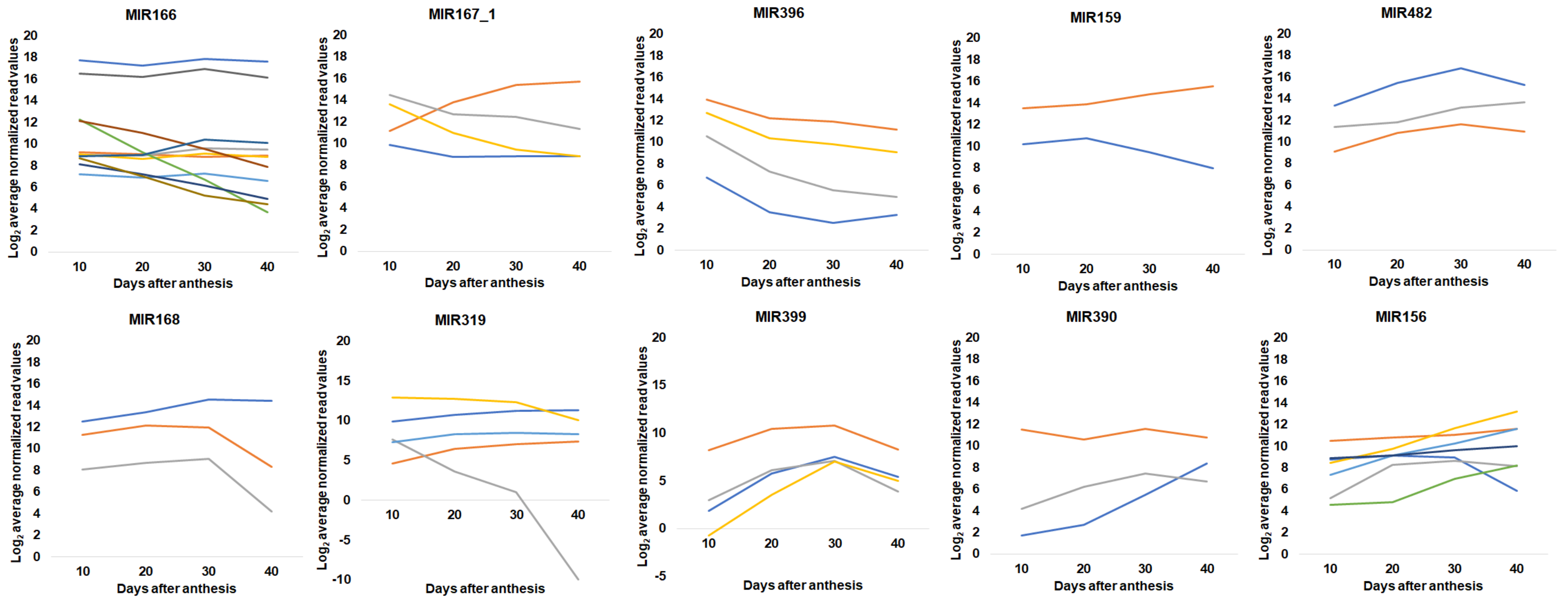

Supplementary Fig. 2: Expression profiles of all expressed miRNAs within selected MIR families during seed development in *Phaseolus vulgaris*. The expression profiles of the miRNAs were obtained using the Log_2 average normalized read values from sRNA-Seq results obtained from samples collected at 10, 20, 30 and 40 days after anthesis.

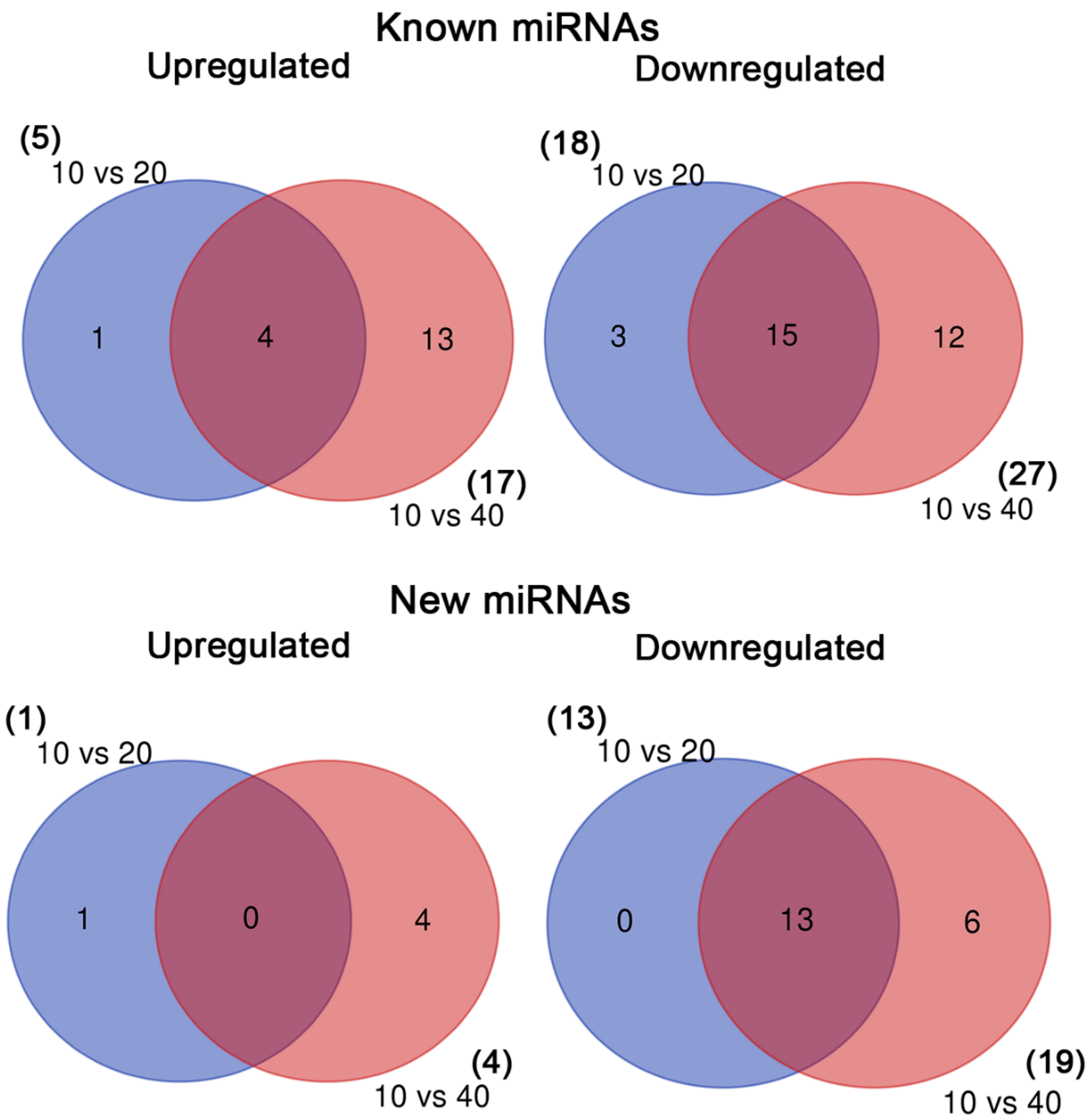

Supplementary Fig. 3: Venn diagrams exhibiting the number of miRNAs differentially expressed (DE) during seed development in *Phaseolus vulgaris*. Venn diagrams were constructed using a freely available web-based tool (http://bioinformatics.psb.ugent.be/webtools/Venn/) with differentially expressed (DE) miRNAs ANOVA and T-test (adj. P ≤ 0.05) between studied timepoints. Since no DE miRNAs were found between 20 vs 30 DAA and 30 vs 40 DAA, they were not included in this analysis. The number of DE miRNAs in each comparison established (10 vs 20 and 10 vs. 40 DAA) are shown between parentheses. (DAA: days after anthesis)

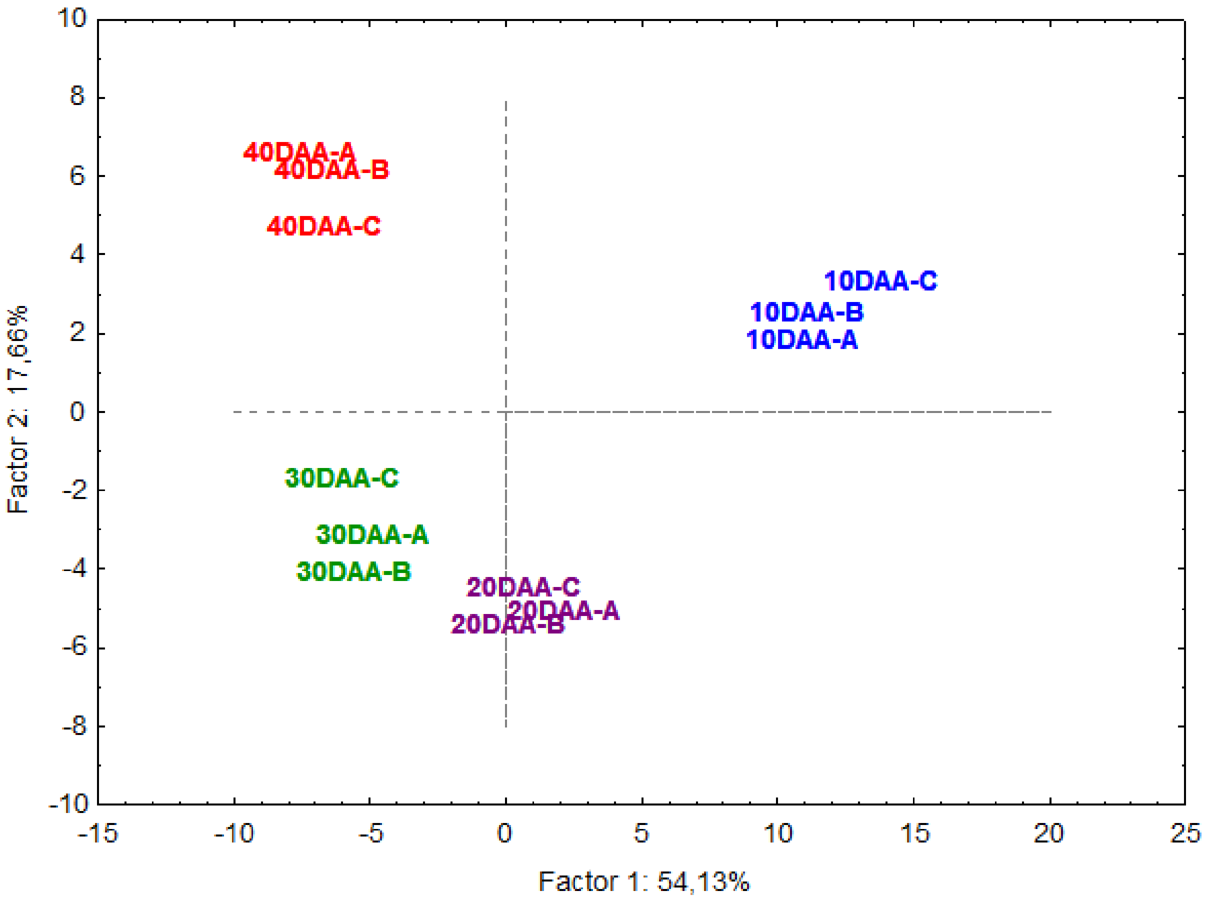

Supplementary Fig. 4: Principal component analysis of all expressed miRNAs during seed development in *Phaseolus vulgaris*. A principal component analysis (PCA) was performed using Statistica, version 6 (Statsoft) and the standardized (Z-score) normalized read values of the expressed miRNAs at 10, 20, 30 and 40 DAA were used as variables. (DAA - Days After Anthesis)

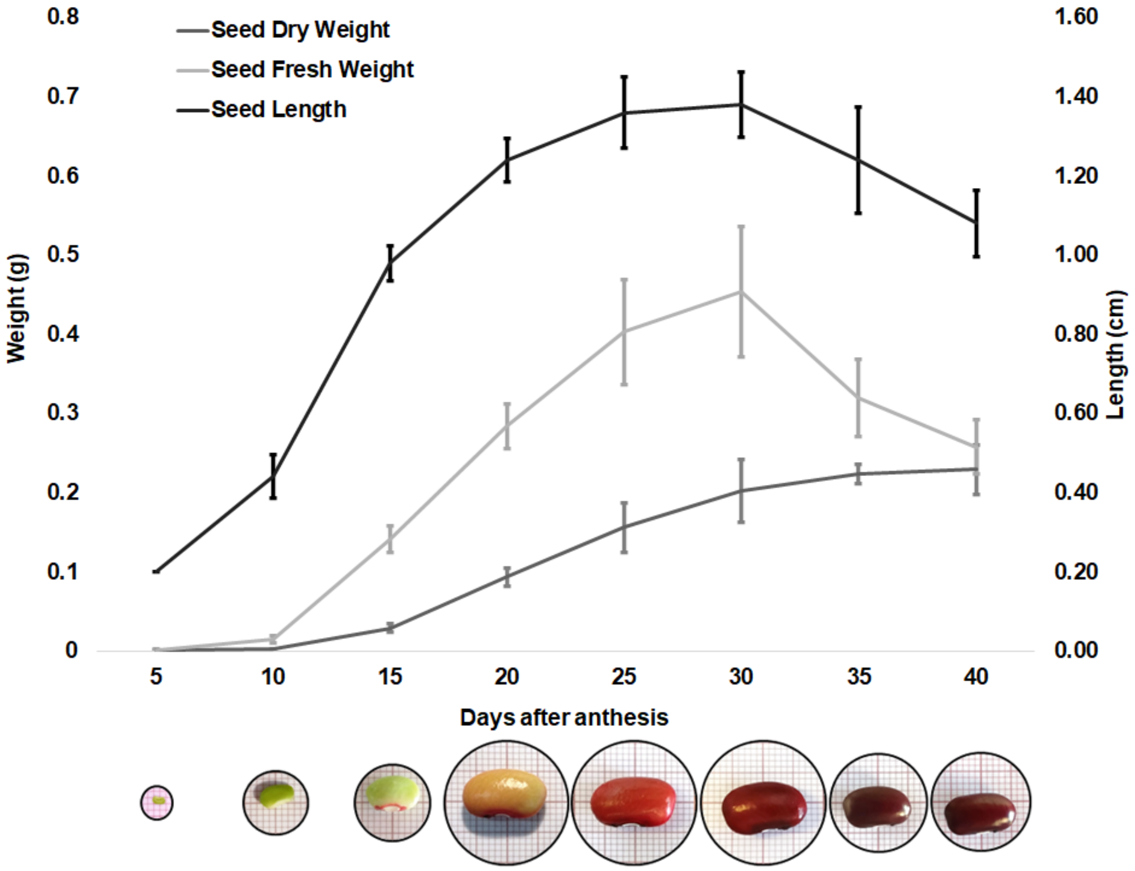

Supplementary Fig. 5: Characterization of the *Phaseolus vulgaris* seed development. Seed morphology, seed fresh weight, dry weight and length were measured at eight consecutive timepoints along the seed development stage studied.

pvu-miR156g_Phvul.005G071500.2:463

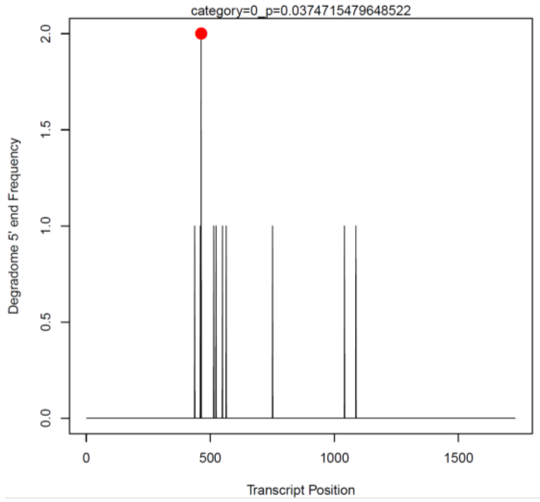

pvu-miR166e_Phvul.002G190000.1:195

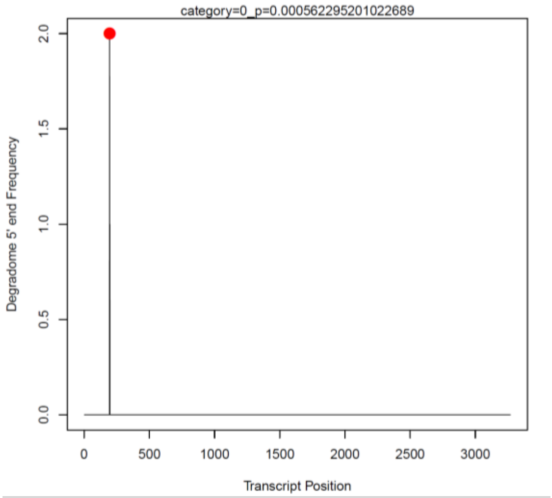

miR_16_Phvul.001G249000.1:1462

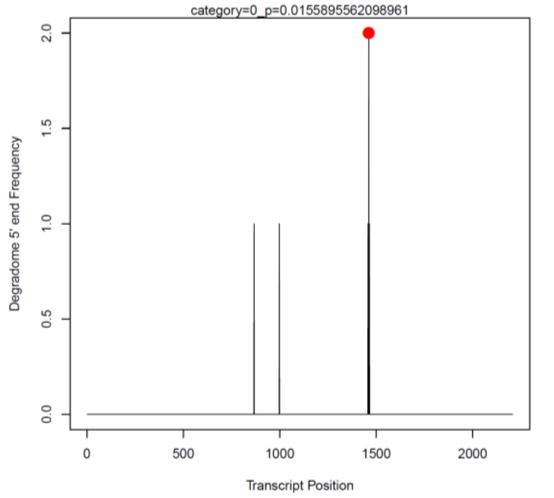

miR_29_Phvul.007G224300.1:1715

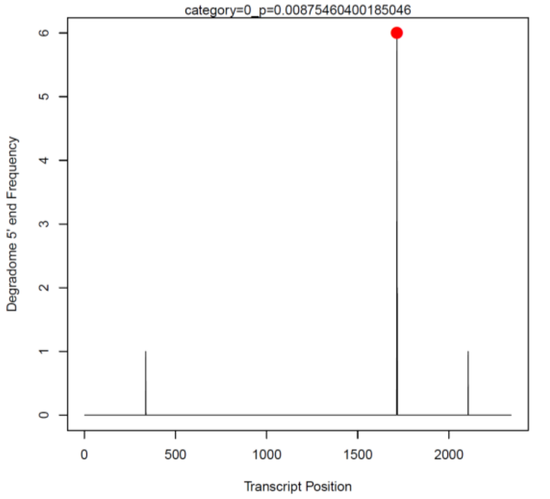

miR_29_Phvul.007G224300.1:1715

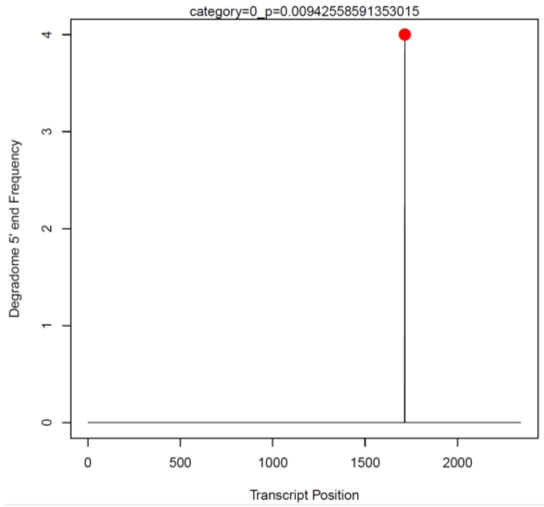

pvu-miR396i_Phvul.008G210300.1:3839

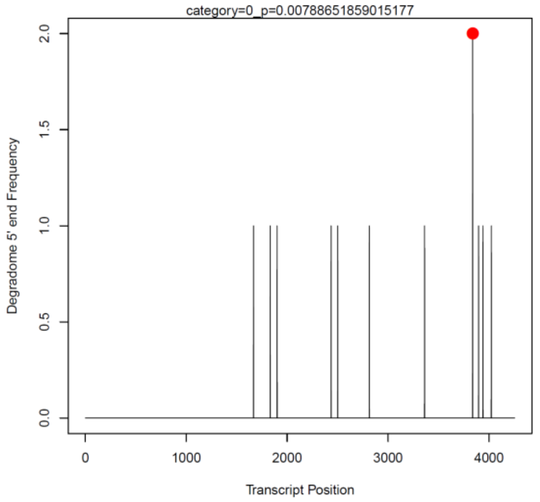

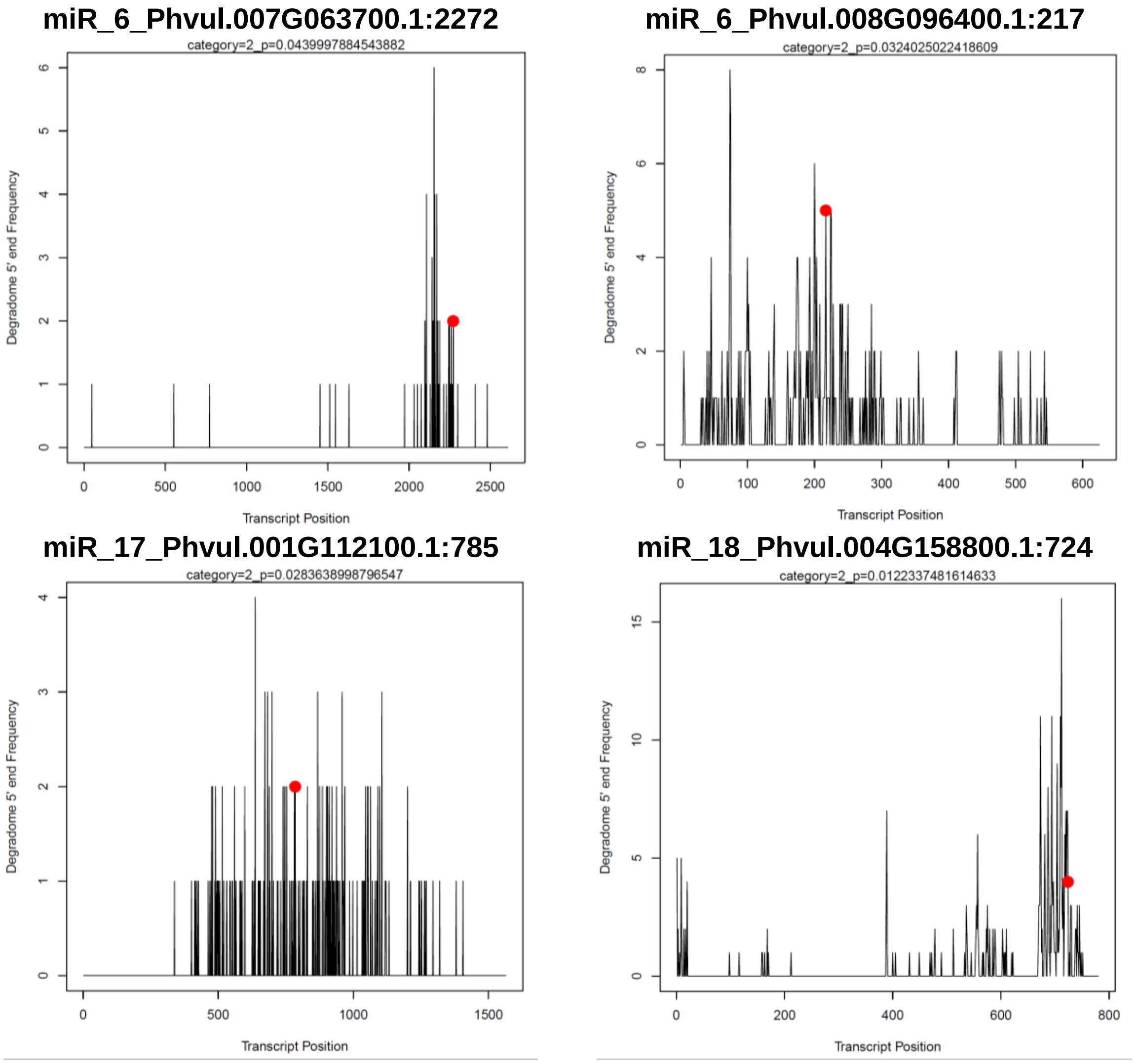

Supplementary Fig. 6: Target plot (t-plots) of representative validated *Phaseolus vulgaris* miRNA targets in different categories as confirmed by degradome sequencing.

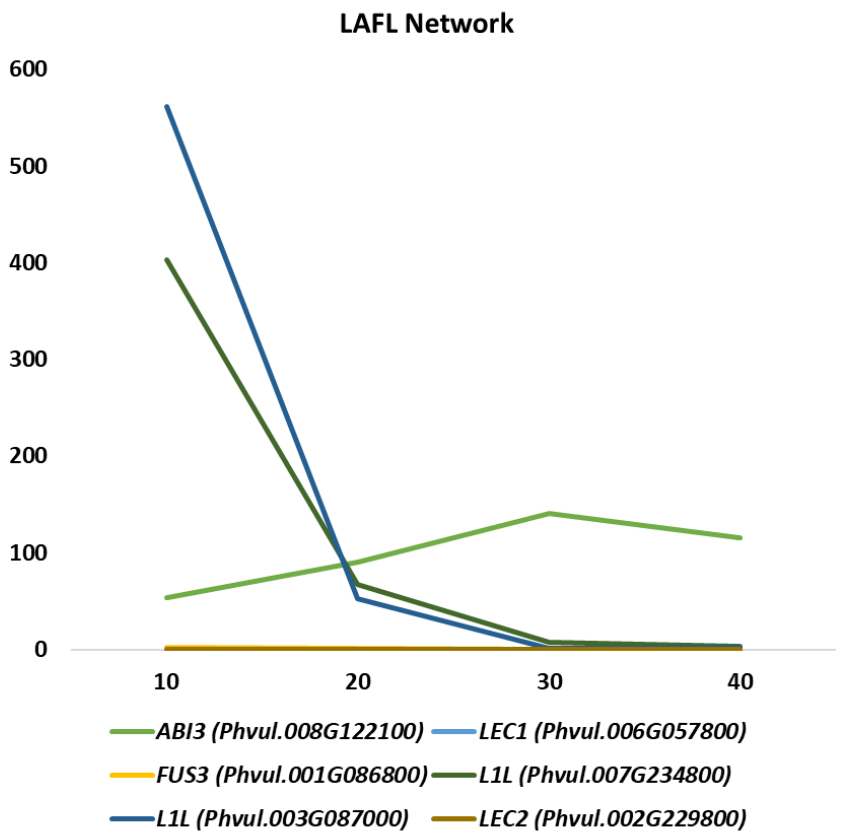

Supplementary Fig. 7: Expression profiles of the members of the LAFL regulatory network during seed development in *Phaseolus vulgaris*. Expression profiles were established using the normalized read value for each target genes at 10, 20, 30 and 40 days after anthesis retrieved from MACE datasets available in Parreira *et al.* (2018). *AP2/B3-LIKE TRANSCRIPTIONAL FACTOR FAMILY PROTEIN* (*ABI3*; Phvul.008G122100); *LEC1 HISTONE SUPERFAMILY PROTEIN* (*LEC1*; Phvul.006G057800); *AP2/B3-LIKE TRANSCRIPTIONAL FACTOR FAMILY PROTEIN* (*FUS3*; Phvul.001G086800); *NUCLEAR FACTOR Y, SUBUNIT B6* (L1L; Phvul.007G234800 and Phvul.003G087000); *AP2/B3-LIKE TRANSCRIPTIONAL FACTOR FAMILY PROTEIN* (*LEC2*; Phvul.002G229800)
